# Are School-Based Interventions Effective in Preventing Internet Misuse? A Systematic Review

**DOI:** 10.1007/s10935-023-00757-9

**Published:** 2023-12-07

**Authors:** Nieves Martínez-Hernández, Daniel Lloret-Irles

**Affiliations:** 1 Department of Health Psychology, University Miguel Hernández, Avinguda de la Universitat d’Elx, s/n, 03202 Elche, Alicante, Spain; 2 Department of Health Psychology, University Miguel Hernández, Faculty of Medicine, 03550 Sant Joan d’Alacant, Alicante, Spain

**Keywords:** Internet addiction, Prevention, Effectiveness, School prevention programs, Adolescent, Internet misuse

## Abstract

Problems related to the use of the internet have triggered the appearance of internet addiction. With this problem comes the need for prevention. The objective of this study is to update the knowledge about the effectiveness of internet addiction prevention programs in schools. The specific aims are: (1) to identify the objectives of school’s prevention programs, (2) to analyse the components and instruments used, (3) to evaluate the quality of the relevant studies and (4) to compare the results of the publications. A search with the terms "Internet", "Internet Use Disorder", "addiction", "prevention", "school", "effectiveness", and "prevention program" was carried out in 10 bibliographic databases in August 2021. For the selection of articles, the 25 quality criteria of the CONSORT 2010 list were used. Only articles that evaluated school prevention programs and had a quality score greater than 10 were selected. The results are shown in three tables. Eleven publications that met the inclusion criteria were identified. The measurement scales used are very heterogeneous. Ten of the 11 programs were effective in preventing adolescent internet misuse, abuse, or addiction. Increasing students' self-control reduces the problem of internet abuse. The validity of the results is discussed considering the methodological quality of the studies.

**Supplementary Information:**

The online version contains supplementary material available at 10.1007/s10935-023-00757-9.

## Introduction

The penetration of screens in everyday life is indisputable, especially among the young and adolescent population, where nine out of ten surf the internet daily (Lenhart et al., 2015). In Europe, approximately 94% of 15-year-olds reported using social networks in the last seven days, with an average time of 2–3 h on school days, although 10% reported spending more than six hours a day online. On nonschool days, the daily average is over five hours. Of those who abuse the Internet, between 4 and 18% will develop an addiction (Sánchez-Carbonell et al., 2008).

Problematic internet use (PIU) is risky, excessive, or impulsive use of the internet that, while not yet addictive, leads to adverse life consequences, specifically physical, emotional, social, or functional impairment, and this is an emerging health concern among adolescents (Moreno et al., 2013). Internet use is increasing every year (Brime et al., 2019). In the Chinese population, the results are alarming, more than 12% of males and 5% of females have symptoms of internet addiction (IA) (Lau, 2011). A study of the prevalence of PIU among university students in Hong Kong appears to be as high as 26.7% (Shek & Yu, 2013). The data collected from the population of Taiwan show that the prevalence of the problem is 1.9% for the general population and 3% for regular internet users (Wu et al., 2019), in Iran, 20% of the population demonstrates PIU (Modara et al., 2017).

In Europe, several studies have shown worrying prevalence rates of PIU. The highest prevalence was found in England (54.9%), followed by Greece (37.7%) and Turkey (32.9%). Furthermore, the results conclude that countries such as Italy, France, Poland, Spain and Hungary have a similar prevalence to each other, that is, 26.4%, 24.8%, 24.5%, 23.7% and 21.6%, respectively (Laconi et al., 2018; Tsitsika et al., 2016).

On many occasions, the main device for internet use is the mobile phone, and its excessive use has been related to high alcohol consumption, tobacco smoking, depression, school failure (Sanchez-Martinez & Otero, 2009), anxiety and insomnia (Jenaro et al., 2007). At the same time, there is a perceived discomfort in these people when they are not online and considerable denial about the existence of the problem (Navarro-Mancilla & Rueda-Jaimes, 2007). In addition, people with behavioural control problems on the internet show four behavioral and emotional components: feelings of anger, depression when they do not have access to the Internet, excessive use of the web and negative repercussions in their daily lives (Aponte Rueda et al., 2017).

Regarding age groups, adolescents are the most vulnerable group to misuse, abuse or become addicted to the internet (Aponte Rueda et al, 2017). Young adults under 21 years of age (Secades-Villa et al., 2014) are at risk due to the slow maturation of areas in the brain involved in behavioural control (Leung, 2017; Casey et al., 2005), and with internet abuse alters their psychosocial development (Tahiroglu et al., 2008). Internet abuse in young people is associated with living outside the family home, low self-esteem and a higher prevalence of problems with other behavioural addictions or substance use (Secades-Villa et al., 2014).

### Prevention

Given the scope and consequences of problematic internet use in the adolescent population, there is a need to develop effective preventive interventions. Consequently, the public administrations have responded both at the regional level (Rioja, 2018; Sánchez Pardo et al., 2018), national level (Ministry of Health, 2020) and European level (Lopez-Fernandez & Kuss, 2019). The schools, due to their ability to reach the adolescent population, are an excellent place to develop universal prevention programs.

The appearance of concern about the impact of information and communication technologies (ICTs) dates back to the beginning of the twenty-first century and since then there have been structured interventions aimed at preventing their misuse (Trahtemberg, 2000).

Despite the intensity and extent of the use of ICTs among the adolescent population, preventive programs whose effectiveness has been evaluated are scarce and, to our knowledge, there are only two previous studies that have reviewed the publications related to the evaluation of the prevention of internet addiction: Vondráčková and Gabrhelík (2016) and Throuvala et al. (2019). They include 21 studies between 2010 and 2017, evaluating different aspects of this topic, but they do not focus solely on studies of the effectiveness of IA prevention programs; they explain the contents of the programs, their characteristics, and the level of acceptance by adolescents. The quality of the studies is also not addressed.

Considering the above, it is necessary to update the knowledge about school prevention programs for internet abuse by adolescents with a systematic review of studies that evaluate the effectiveness of preventive interventions for AI, (Sánchez-Meca et al., 2010). For this work, the following specific aims were established: (1) identify the objectives of prevention programs taught in schools, (2) analyse the components and instruments used to verify that the results of the different articles can be compared; (3) evaluate the quality of published studies and (4) compare the results of these publications.

## Methods

### Procedure, Inclusion and Exclusion Criteria

In response to the above objectives, a systematic review was developed and a search strategy devised to examin original and quantitative studies with the objective of evaluating the efficacy of a preventive intervention for the problematic or addictive use of the internet; the PRISMA checklist can be found in Appendix 1 of supplementary information (Page et al., 2021)-. The inclusion criteria were of two types: quality criteria and content or theme criteria. Regarding the quality criteria, the CONSORT 2010 quality checklist (Cobos-Carbo & Augustovski, 2011) was used with 25 points divided into 6 sections: title and abstract, introduction, methods, results, discussion and other information. The criteria were that the selected articles had to score more than 10 on the CONSORT 2010 quality checklist. Due to the overall quality of the studies retrieved in the database search, we decided to assume a score of 10 as the inclusion criterion, to prevent dropout. In terms of content, the articles had to evaluate an abuse prevention program or IA in the school setting.

The search was performed in ten databases (PubMed, Scopus, Health Evidence, Agency for Healthcare Research and Quality, Guidelines International Network, ERIC, WHO, HNT and Cochrane). The following keywords were used: "Internet", "Internet Use Disorder", "addiction", "prevention", "school" "effectiveness" "prevention program", no time filters were applied and the titles, abstracts and keywords were reviewed. The combinations used were: “Internet AND addiction AND prevention”, “Internet AND addiction AND prevention AND school”, “Internet AND prevention AND school”, “internet AND addiction AND prevention AND effectiveness” and “internet addiction AND prevention program”. Table 1 shows the results obtained in each of the databases.

**Table 1:** Number of results per database

Database	Results	Date
PubMed	73	07/2021
Scopus	172	05/2021
Health Evidence	47	04/2021
Agency for Healthcare Research and Quality	0	04/2021
Guidelines International Network	0	04/2021
ERIC	15	07/2021
QUIEN	15	07/2021
HNT	0	06/2021
Cochrane	56	08/2021
TOTAL	378	

The selection of publications for the review was organized into three phases.

Phase 1 was the primary search performed through search engines, which found 391 publications.

Fifty-nine studies were eliminated due to duplication. The titles of the remaining 332 studies were reviewed, and 198 publications were discarded by the main researcher for not meeting the inclusion requirements related to content: the articles did not evaluate an abuse prevention program or IA in the school setting.

In Phase 2, the remaining 134 abstracts were analysed by the main researcher, who evaluated the closeness of the topic of the publication with the objectives of this work. Six were eliminated because they were not in English or Spanish, 107 because they evaluated the need for a prevention program and not the effectiveness of a program. Seven of the articles were eliminated because they were systematic reviews and another five did not meet the quality criteria, leaving nine publications for the last phase.

In Phase 3, the full text of the remaining nine publications was read by both researchers independently, and all nine met the inclusion criteria.

**Fig. 1:**
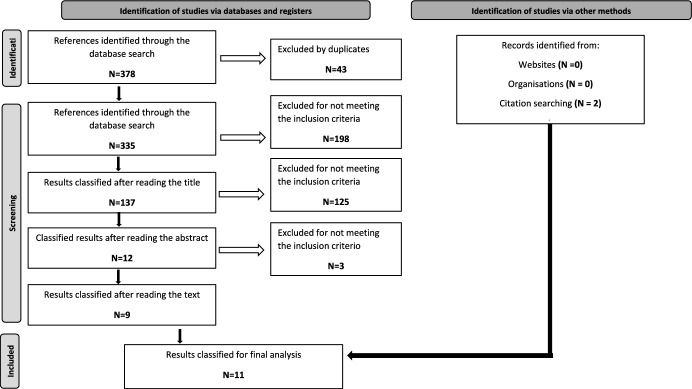
Prisma’s flowchart of bibliographic search strategy

During the selection process, two additional articles were identified in the bibliographic references of the reviewed publications. Therefore, the final number of studies included in this review is 11. The outline of the search process is shown in Fig. 1.

In response to the third objective, to determine the quality of the studies, 25 quality criteria were defined from the CONSORT 2010 quality checklist. It was considered that the articles had a control group, that they used repeated measures postintervention at least six months later and that they had random samples. The score of each article was calculated by fulfilling the six indicators in which the quality criteria are grouped (see Appendix 2 of supplementary information).

The assessment of bias in the articles included in this review was carried out using the three factors included in the PRISMA system: whether the instruments used were validated tools, whether the article used external reviewers and the number of external reviewers (Page et al., 2021). For the first factor, three possible scores were given: 0 if the paper did not include any validated tools, 1 when some of the instruments contained validated scales, and 2 when all the scales used were validated. All articles scored 2 on this scale except the papers by Walther et al. (2014), Shek and Yu, (2011) and de Leeuw et al. (2010) which scored 1 because they used some ad hoc questionnaires. The second factor, internal or external reviewers, scored 0 for articles with only internal reviewers and 1 for those with external reviewers. Only the paper by Du et al. (2010) scored 1, as it had two psychiatrists who reviewed the training and application of the teachers. It should be noted that the study by de Leeuw et al. (2010) also supervised the training of the teachers, but the research team conducted the training. Finally, the number of reviewers (internal or external) was noted. The work by Çelik, (2016) had one; and the papers by Li et al. (2017), Busch et al. (2013), de Leeuw et al. (2010) and Hou et al. (2019) had three. The remaining papers had two. Therefore, a higher score means a greater effort was made to control bias. The sum of the scores for each article can be found in Table 2.

**Table 2:** Description of the efficacy evaluation studies

No	Author (year)	Title	Country	Program (Authors)	Design	Sample	Quality
1	Li et al. (2017)	A 2-year longitudinal psychological intervention study on the prevention of Internet addiction in junior high school students of Jinan city	China	Own program: (Li et al.)	Longitudinal 24 months Random: Yes Cntl.Gr: Yes Eva. internal	N = 759 students Gr Exp. n = 446. Cntl.Gr. n = 385 Hometown: 1 Jinan City High School Inclusion criteria: no alterations in IADDS and Symptom Checklist 90 SCL-90	22
2	Uysal and Balci, (2018)	Evaluation of a School-Based Program for Internet Addiction of Adolescents in Turkey	Turkey	Healthy Internet Use Program (Uysal & Balci)	Longitudinal 9 months Random: No Cntl.Gr: Yes Eva. internal	N = 64 16–18-year-olds from two high schools Gr Exp. n = 41, Cntl.Gr. n = 43 Inclusion criteria: > 91 in The Internet Addiction Scale	13
3	Yang and Kim, (2018)	Effects of a prevention program for Internet addiction among middle school students in South Korea	South Korea	Own program: (Yang & Kim)	Cross Random: No Cntl.Gr: Yes Eva. internal	N = 79 8th grade students between 13–15 years old from two secondary schools Gr Exp. n = 38, Gr. Cntl. n = 41 Inclusion criteria: score more than 108 on Internet Addiction propensity Scale	21
4	Walther, Hanewink, & Morgenst (2014)	Effects of a Brief School-Based Media Literacy Intervention on Digital Media Use in Adolescents: Cluster Randomized Controlled Trial	Germany	Vernetzte www.Welten Walther et al. (2014)	Longitudinal 12 months Random: Yes Cntl.Gr: Yes Eva. internal	N = 1,843 12-year-olds from 27 schools Gr Exp. N = 922 Gr. Cntl. N = 921 Inclusion criteria: have parental permission, be present on the day of data collection	21
5	Kormkaz & Kiran-Esen, (2012)	The Effects of Peer-Training about Secure Internet Use on Adolescents	Turkey	Peer Training Program. National Peer Helpers Association, (2002)	Cross Random: No Cntl.Gr: Yes Eva. external	N = 825 6th, 7th and 8th grade students from primary schools in Mersin (Turkey) between 13 and 15 years old exp group _ n = 410 Group cont. n = 415 Inclusion criteria: have parental permission	12
6	Shek and Yu, (2011)	Prevention of Adolescent Problem Behaviour: Longitudinal Impact of the Project PATHS in Hong Kong	Hong Kong	Project PATHS (Positive Adolescent Training through Holistic Social Programs), Shek & Yu	Longitudinal 36 months. Random: Yes Cntl.Gr: Yes Eva. internal	N = 7846 at the beginning of the process from 48 Hong Kong secondary schools group count n = 3797 Group exp. n = 4049	23
7	Busch et al. (2013)	Results of a Multibehavioral Health-Promoting School Pilot Intervention in a Dutch Secondary School	Netherlands	Applied Health Promoting School principles (Rasmussen & Rivett)	Longitudinal 36 months Random: No Cntl.Gr: No Eva. external	336 students from 15 to 16 years old (4th grade)	11
8	Celik (2016)	Educational intervention for reducing Internet addiction tendencies	Turkey	Training-Program Sessions to Increase Conscious Internet Use, Academic Motivation, and Efficient Use of Time. (Celik)	Longitudinal 6 months. Random: Yes Cntl.Gr: Yes Eva. internal	N = 30 high school students exp group _ n = 15 Group Cont. n = 15 Inclusion criteria: students with scores higher than the mean of the sample in the PIUS	18
9	de Leeuw et al. (2010)	Internet and game behaviour at a secondary school and a newly developed health promotion programme: A prospective study	countries Low	The media literacy of children and young people. Buckingham (2005)	Longitudinal 12 months Random: No Cntl.Gr: No Eva. external	475 high school students aged 11–18. That they were present on the days of carrying out the pre- and post-treatment tests	16
10	Du et al. (2010)	Longer term effect of randomized, controlled group cognitive behavioural therapy for Internet addiction in adolescent students in Shanghai	Shanghai	Cognitive behavioural therapy group	Longitudinal 6 months Random: Yes Cntl.Gr: Yes Eva. external	56 students with internet addiction according to Beard and Wolf criteria, with a mean of 16.63 ± 1.23 years exp group _ n = 32 Group Cont. n = 24	19
11	Hou et al. (2019)	Social media addiction: Its impact, mediation, and intervention	China	Ad hoc intervention program with cognitive restructuring for addiction to social networks based on the work of Young's	Cross Random: Yes Cntl.Gr: Yes Eva. internal	38 students from Peking University with a mean age of 19.71 ± 1.43 years Exp.Gr n = 18 Cnt.Gr n = 20	10

## Results

Eleven studies were analysed. The basic data of the studies are shown in Table 2.

In response to the first objective, the purposes of prevention programs are identified. In all programs the purpose is related to the use of the internet by adolescents. Program objectives are described in more detail in Table 3. The most frequent purpose was to increase students' self-control, followed by work on healthy internet use habits, reduce internet use and prevent internet addiction. Each of the studies attempted to achieve its objective through different activities. P.A.T.H.S. is the program with the most components (Shek & Yu, 2011), including bonding, resilience, social competence, recognition of positive behaviour, competence (emotional, cognitive, behavioural and moral), self-determination, self-efficacy, clear and positive identity, focus on the future, prosocial participation and prosocial norms. Several authors intended to achieve their goals by working on the habits and behaviours of a healthy life in relation to internet use (Busch et al., 2013; Du et al., 2010; Li et al., 2017; Uysal & Balci, 2018; Walther et al., 2014; Yang & Kim, 2018) or caring for the mental health of adolescents (Du et al., 2010; Hou et al., 2019; Li et al., 2017). Other authors aimed to increase self-regulation and self-efficacy skills (Yang & Kim, 2018), self-reflection or self-control (Du et al., 2010; Walther et al., 2014). Two other studies focused on improving academic motivation (Çelik, 2016; Hou et al., 2019). The article by de Leeuw et al. (2010) also takes into account in its intervention other factors of internet use such as digital communication, online bullying, digital image, online sexuality and distorted ideals of beauty. Two authors also examined work on intrapersonal concepts through peer training (Kormkaz & Kiran-Esen, 2012) or by increasing parents' awareness of the problem (Uysal & Balci, 2018).

**Table 3:** Description of prevention programs

*n*o	Program	Goals	Results	Variable	Instrument
1	N. Sessions: 5 in each block Frequency: 1 block at the beginning of each semester Duration: 1 h Facilitator: psychiatrists and teachers Techniques: psychoeducation	-Improve mental health -Prevent internet addiction	⇑Mental health ⇓Internet addiction	IA Severity General symptoms	IADDS (Zan et al., 2008) SCL-90 (Wang et al., 1999)
2	N. Sessions: 8 Frequency: no data Duration: between 40 and 80 min Facilitator: psychologist Techniques: watch movies, play games, summarize, draw, make posters, organize and use flashcards	- Reduce the use of the Internet - Develop healthy use habits - Learn to better organize daily life activities - Raise parents' awareness of healthy Internet use by children	⇓Internet addiction compared with the measurements made at 3 and 9 months later	IA Severity	IAS (Nicholas & Niki, 2004) Interview with parents Weekly calls to parents
3	N. Sessions: 10 Weekly frequency Duration: 45 min/ week Facilitator: School Nurse Techniques: psychoeducation, role playing, group activities and written tasks	-Work on self-efficacy and self-regulation -Study the effect of these skills on Internet use time, Internet addiction and self-control	⇑Self-regulation and self-efficacy - Internet usage time Exp.Gr < Cntl.Gr - Internet addiction Exp.Gr < Cntl.Gr Self-regulation, self-efficacy and self-control predictors of problematic Internet use	Self-control Self-efficacy IA Severity	Brief-Self Control Scale (Cho, 2011) Self-Efficacy Scale (Kim, 2001) K-Scale. Internet Addiction Prone Scale (Agency, 2003)
4	N. Sessions: 4 Weekly frequency Duration: 90 min/ session Facilitator: trained teacher Techniques: psychoeducation, self-observation, debate and student reflection	-Work on students' self-reflection and self-control to reduce the problematic use of the Internet and reduce computer games	⇓Time spent on games Internet abuse and addiction Exp.Gr = Cntl.Gr	Severity addiction video game IA	KFN-CSAS-II (Rehbein et al., 2010) ISS (Hahn & Jerusalem, 2001) Observation of parents and house rules Data sociodemographic
5	N. Sessions: 10 Weekly frequency Duration: 90 min Dynamizer: prepared adolescents Techniques: psychoeducation and group activities	- Work on skills related to the Internet through peer training	Internet usage time Exp.Gr < G. Cntl Quality of Internet use. Exp.Gr > G. Cntl	Internet usage habits	The Internet Use Habit Scale (Yılmazhan-Gültutan, 2007)
6	N. Sessions: 40 per course Frequency: bi-weekly Duration: at least 30 min/ session for 3 courses Dynamizer: prepared professionals Techniques: holistic social programs	- To study the longitudinal effects of the PATHS program in the prevention of problem behaviours in young people	control and self-control Exp.Gr > G. Cntl Internet addiction and abuse Exp.Gr < G. Cntl	Self-monitoring on the internet Positive Youth Development Measure	Internet Use Control Chinese Positive Youth Development Scale (CPYDS) (Shek & Ma, 2010)
7	N. Sessions: 30 per course Weekly frequency Duration: 50 min Facilitator: trained teacher Techniques: psychoeducation and group activities	- Give tools to students enable them to make healthy choices - Reduce excessive compulsive behaviour related to internet use and gaming	Compulsive internet use ⇓Women = Males video game addiction No significant changes	Health survey Socioeconomic status Prosocial behaviour Compulsive internet use Video game addiction	Dutch Health Behaviour in School-aged Children (HBSC) The Family Affluence Scale (FAS) (Boyce et al., 2006) Strengths and Difficulties Questionnaire (SDQ) (Goodman et al., 1998) Compulsive Internet Use Scale (CIUS) (Meerkerk et al., 2009) Videogame Addiction Test (VAT) (Derived from CIUS)
8	N. Sessions: 5 Weekly frequency Duration: from 90 to 120 min Facilitator: trained teacher Techniques: psychoeducation	- Reduce the time that study group members spent on the Internet - Improve awareness by increasing your academic motivation - Reduce internet addiction	Internet addiction Exp.Gr < G. Cntl academic motivation Exp.Gr > G. Cntl	Problematic internet use	The Problematic Internet Use Scale (PIUS) (Ceyhan et al., 2007)
9	N. Sessions: 64 Frequency: bi-weekly Duration: 1 h Facilitator: trained teacher Techniques: psychoeducation	- Prevent internet addiction and online games	⇓Online gaming users ⇓Heavy internet use	Compulsive internet use Compulsive use of online gambling prosocial behaviour Socioeconomic status	The Compulsive Internet Use Scale (CIUS) (Meerkerk et al., 2009) Compulsive Game Use Scale (CGUS) (Adaptation of CIUS) Strengths and Difficulties Questionnaire (SDQ) (Goodman et al., 1998) Family Affluence Scale (FAS) (Boyce et al., 2006)
10	N. Sessions: 8 Weekly frequency Duration: between 1.5 h and 2 h Facilitator: 2 psychiatrists Techniques: Cognitive behavioural group therapy and psychoeducation	- Reduce internet usage - Increase time management skills, emotional and cognitive management - Improve emotional state	⇓Internet use Exp.Gr = Cntl.Gr Time management skills, emotional management Gr. Exp > G. Cntl Improved emotional state and self-management Exp.Gr > Cntl.Gr _	IA Severity of abusive internet use Perceived control of free time prosocial behaviour	Beard's Diagnostic Questionnaire for Internet addiction (Du et al., 2008) Internet Overuse Self-Rating Scale (Cao et al., 2006) The Time Management Disposition Scale (TMDS) (Huang & Zhang, 2001) Strength and Difficulties Questionnaire (Chinese edition) (Goodman et al., 1998)
11	N. Sessions: 4 Weekly frequency Duration: 1 h Facilitator: psychologist Techniques: Cognitive restructuring	- Reduce addiction to social networks - Improve the mental health of students - Increase your academic efficiency	Internet and social media addiction Exp.Gr < Cntl.Gr ⇑Mental health and academic efficiency	Social media addiction	Bergen Social Media Addiction Scale (BSMAS) (Lin et al., 2017) Ad hoc questionnaire on hours of sleep, hours of internet use

⇑ = Increases; ⇓decreases; = There are no differences

Exp.Gr. = Experimental Group Cntl.Gr = Control Group

The second objective, was to analyse the components and instruments used (see Table 3). None of the 11 programs used the same measurement instruments; however, in the articles by Busch et al. (2013) and Leeuw et al. (2010), the Family Affluence Scale (FAS), which measures the family's socioeconomic level, and the Strengths and Difficulties Questionnaire (SDQ), which assesses hyperactivity-inattention, emotional symptoms, problems with peers, behavioural problems, and prosocial behaviour, were repeated.

The methodology varied greatly. All the studies include several sessions that can vary between 4 and 64 with a duration and periodicity that are also variable, but not greater than 2 h, and with a frequency that is generally weekly. Regarding the design, three studies are cross-sectional and eight longitudinal with measurements from 6 to 36 months. Regarding the variables that the studies measure, the most common is IA or its severity, followed by the habits of using the internet and social networks and the use and abuse of online video games, but general psychological symptomatology is also measured, such as self-control, self-efficacy, youth development, general health, social context, socioeconomic status, and time management.

The third objective, to evaluate the quality of the published studies, it is reflected in Table 2 and in Appendix 2 of supplementary information. The quality evaluation followed the CONSORT 2010 methodology (Cobos-Carbo & Augustovski, 2011) which has 25 points divided into 6 sections: title and abstract, introduction, methods, results, discussion, and other information. Only four studies mention the theoretical basis for the quality evaluation: Çelik (2016); Hou et al. (2019); Shek and Yu, (2011); Uysal and Balci (2018). All of the publications clearly define the program population and the participant retention rate.

Regarding whether the studies evaluate their own program or another program, there are both types in this review. The studies that evaluate an external program are: Busch et al. (2013); de Leeuw et al. (2010); Du et al. (2010) and Kormkaz & Kiran-Esen, (2012).

Four of the studies carried out the evaluation in a sample size greater than 500 (Li et al., 2017); Walther et al., 2014; Kormkaz & Kiran-Esen (2012); Shek and Yu (2011). Regarding the design, three studies have only prepost measurement because they are cross-sectional: Hou et al. (2019); Kormkaz & Kiran-Esen, (2012) and Yang and Kim, (2018). Regarding whether they have a control group, only two of the articles have a design without this group: Busch et al. (2013) and de Leeuw et al. (2010).

The fourth objective was to compare the results of the publications studied, which can be seen in Table 2. All intervention programs sought to reduce misuse, abuse, or IA in young people. All of them, except Walther et al. (2014) and Busch et al. (2013), who did it only in the female sample, achieved it.

All other studies were successful in lowering scores related to internet abuse. Among the results, an improvement in the mental health of the students stands out (Hou et al., 2019 and Li et al., 2017), the significant increase in the level of self-regulation and self-efficacy (Yang & Kim, 2018), more adapted use of the internet (Kormkaz & Kiran-Esen, 2012), the acquisition of control and self-control skills (Du et al., 2010 and Shek & Yu, 2011), the increase in academic efficiency (Çelik, 2016 and Hou , et al., 2019), improved time management (Du et al. (2010)) and reduced use of online games (Busch et al., 2013; de Leeuw et al., 2010 and Walther et al., 2014) In relation to the duration of the effects, all the longitudinal articles found that the efficacy of the intervention was maintained during the months in which they continued to follow up.

## Discussion

The systematic review focused on the comparison of the objectives of each study, its components and instruments, the quality of each of the publications and the results obtained.

Previous reviews collected a total of 21 studies between the years 2010 and 2017 (Throuvala et al., 2019; Vondráčková & Gabrhelík, 2016). In our work, the review period extended to 2020 and three publications after 2017 are provided (Li et al., 2017; Uysal & Balci, 2018; Yang & Kim, 2018). In addition, an analysis of the quality of the studies was carried out, thus correcting a limitation of the previous reviews.

Most of the studies conclude that the evaluated programs are effective in terms of the problem of misuse, abuse or IA. It is true that the methodological variability between programs is wide.

### Healthy Habits and Behaviours

The programs that worked on healthy habits and behaviours in the use of the network significantly reduced IA scores (Busch et al., 2013; de Leeuw et al., 2010; Shek & Yu, 2011; Uysal & Balcí, 2018). This indicates that adolescents, became more aware of what it means to have a healthy relationship to ICTs and increasing face-to-face social relationships as they felt less need to connect to the network.

In the programs of Du et al. (2010), Hou et al. (2019) and Li et al. (2017) the authors found that after group psychoeducational intervention sessions aimed at reinforcing emotional management skills, the use of ICTs decreased, which supports the relationship between mental well-being and adapted use of the internet.

### Skills and Competencies

The Healthy Internet Use Program (Uysal & Balci, 2018), in addition to working on healthy habits, opted to reinforce daily life skills and involve parents in this process. The significant improvement lasted, at least nine months after the implementation of the project. This indicates that psychoeducation given to parents can contribute to prolonging the beneficial effects on their children.

The training of self-efficacy, self-regulation and self-control skills was the objective of the program implemented by Yang and Kim (2018), who verified that the higher the score in self-regulation and self-efficacy, the lower the scores in internet usage time and addiction. This confirms that poor management of these skills is a predictor of misuse of the internet.

However, the results of the evaluation of the Vernetzte www.Welten program that uses the same variables does not result in improve IA scores, although it does in the parameters of video game behaviour (Walther et al., 2014). This suggests that the digital behavior of adolescents is not uniform.

Korkmaz and Kiran-Esen (2012) opted for peer training. They worked on aspects of communication skills, self-knowledge, public speaking, knowledge of relating to ICT. The results demonstrate a significant reduction in the time of internet use and the quality of its use in the experimental group. Future research, could evaluate whether peer training produces different effects than programs taught by adult professionals.

The P.A.T.H.S. program is the most complete of those analysed in this work. It works on 15 youth development constructs, and their influence on many problem behaviours (Shek & Yu, 2011). In this analysis, only the data referring to the excessive use, abuse and IA of the participants were taken into account. The students in the experimental group acquired self-control skills thanks to which the problematic use of the internet was reduced compared to the control group.

### Academic Motivation

Çelik's (2016) approach focused on academic motivation, a factor that is also included in the work of Hou et al. (2019). The hypothesis was that if students increased their study time, this would reduce their time spent on the internet. Both succeeded, especially Çelik (2016) with his ad hoc program specifically designed for this purpose.

Regarding the quality of the study, it should be noted that the four studies with the highest quality according to CONSORT 2010 (Cobos-Carbo & Augustovski, 2011) with a score greater than 20 are: Li et al. (2017), Shek & Yu (2013), Walther et al. (2014) and Yang and Kim (2018). In all these studies, positive results were achieved in the reduction of IA in addition to an increase in mental health in Li et al. (2017), greater self-regulation in (Yang & Kim, 2018) and greater self-control in Shek & Yu (2013). However, the study by Walther et al. (2014), despite having a quality of 21 according to the CONSORT 2010 criteria, does not find conclusive results regarding abusive use or IA, although there is a significant decrease in the time of internet use.

Internet abuse prevention programs should be more specific in terms of their content, that is, they should differentiate between social networks, video games, internet searches, online school tasks, etc. The low specification of internet use that they seek to reduce or prevent, makes the results difficult to interpret. Multicomponent programs have been shown, in this and other systematic reviews, to be more effective in preventing abuse and addiction to the internet. Likewise, the most dynamic and participatory programs have also obtained better results. (Espada Sánchez et al., 2015; Faggiano et al., 2014).

### Limitations

There are limitations related to the languages of the publications. Although only articles written in English were analysed, only 2 efficacy evaluation studies were discarded because they were written in other languages (Korean). This makes us think that production in other languages is scarce. Of the 11 studies analysed, 10 come from non-English speaking countries.

Another limitation is the existence of publication bias, which consists of the editorial tendency to publish mostly significant results to the detriment of investigations that report a nonsignificant relationship between the variables under investigation. This poses a risk of overestimating the efficacy and effectiveness of interventions and policies to prevent internet over use.

The fact that there is no consensus on the use of the measurement instrument is also a very significant limitation. Therefore, the comparison of the objective concept, that is, the IA, cannot be guaranteed to be totally objective.

However, the rate of inbreeding is high, and 7 of the 11 studies evaluated their own prevention program, which threatens the validity of the results, especially when they are positive.

It should be mentioned that there are aspects of the publications to improve in future lines of research. More longitudinal studies are needed (Brime et al., 2019) which would make it possible to identify what type of prevention has lasting effects on young people.

We trust that the results of this work will facilitate the work of professionals when choosing a suitable program for their target population, knowing its possible limitations. At the same time, knowing the objectives of each of the programs, it might be possible to develop a new program that encompasses the greatest number of strengths from each of the studies.

## Conclusions

Almost all studies have shown good efficacy in preventing misuse, abuse, and IA. The objectives of the programs are to improve self-control skills, self-knowledge, self-regulation, self-efficacy, organization of free time and daily responsibilities, thereby improving youths’ mental health in general. The average number of sessions of a preventive program was 18, with programs ranging from 4 to 64 sessions. Three studies presented a program developed by different authors to those of the analysed article.

The severity measure of IA is highly variable, which makes it difficult to compare the results. Authors from different countries use very different instruments to measure the same phenomenon. It seems that there is no general way to assess IA because there is a need to validate such an instrument for each population. At the same time, the different programs relate IA to different behavioural aspects and to different cognitive abilities.

### Supplementary Information

Below is the link to the electronic supplementary material.

Supplementary file1 (DOCX 151 KB)

## References

[CR1] Agency, N. I. (2003). *Internet addiction self‐report questionnaires (K‐tool) and prevention and education program.* Seoul: Korea Agency for Digital Opportunity and Promotion.

[CR4] Aponte Rueda DR, Castillo Chávez P, González Estrella J (2017). Prevalencia de adicción a internet y su relación con disfunción familiar en adolescentes. Revista Clínica de Medicina de Familia.

[CR7] Boyce W, Torsheim T, Currie C, Zambon A (2006). The family affluence scale as a measure of national wealth: Validation of an adolescent self-report measure. Social Indicators Research.

[CR8] Brime, B., Llorenes, N., Méndez, F., Molina, M., & Sánchez, E. (2019). *Informe sobre adicciones comportamentales.* Observatorio Español de las Drogas y las Adicciones Delegación del Gobierno para el Plan Nacional sobre Drogas.

[CR90] Buckingham, D. (2005). *Educación en medios*. Barcelona: Paidós.

[CR9] Busch V, De Leeuw RJ, Schrijvers AJ (2013). Results of a multibehavioral health-promoting school pilot intervention in a Dutch secondary school. Journal of Adolescent Health.

[CR10] Cao F, Su L, Gao X (2006). The related factors to internet overuse in middle school students. Chinese Journal of Psychiatry.

[CR11] Casey B, Tottenham N, Liston C, Durston S (2005). Imaging the developing brain: What have we learned about cognitive development?. Trends in Cognitive Sciences.

[CR12] Çelik ÇB (2016). Educational intervention for reducing Internet addiction tendencies. The Turkish Journal on Addiction.

[CR13] Ceyhan E, Ceyhan AA, Gürcan A (2007). Problemli İnternet Kullanımı Ölçeği’nin geçerlik ve güvenirlik çalışmaları. Kuram Ve Uygulamada Eğitim Bilimleri.

[CR14] Cho HI (2011). The effect of high school students’ perceived time perspective on self-regulated learning: Self-control as a mediator. Future Oriented Youth Society.

[CR15] Cobos-Carbo A, Augustovski F (2011). Declaración CONSORT 2010: Actualización de la lista de comprobación para informar ensayos clínicos aleatorizados de grupos paralelos. Medicina Clínica.

[CR16] de Leeuw JRJ, de Bruijn M, de Weert-van Oene GH, Schrijvers AJ (2010). Internet and game behaviour at a secondary school and a newly developed health promotion programme: A prospective study. BMC Public Health.

[CR17] Du Y, Kou J, Coghill D (2008). The validity, reliability and normative scores of the parent, teacher and self report versions of the Strengths and Difficulties Questionnaire in China. Child and Adolescent Psychiatry and Mental Health.

[CR18] Du YS, Jiang W, Vance A (2010). Longer term effect of randomized, controlled group cognitive behavioural therapy for Internet addiction in adolescent students in Shanghai. Australian & New Zealand Journal of Psychiatry.

[CR21] Espada Sánchez, J. P., Gonzálvez Maestre, M. T., Orgilés Amorós, M., Lloret Irles, D., & Guillén Riquelme, A. (2015). Meta-analysis of the effectiveness of school substance abuse prevention programs in Spain. *Psicothema*, *27*(1). 10.7334/psicothema2014.106 25633763

[CR22] Faggiano F, Minozzi S, Versino E, Buscemi D (2014). Cochrane database of systematic reviews. Cochrane Database of Systematic Reviews.

[CR25] Goodman R, Meltzer H, Bailey V (1998). The Strengths and Difficulties Questionnaire: A pilot study on the validity of the self-report version. European Child & Adolescent Psychiatry.

[CR28] Hahn A, Jerusalem M, Theobald A (2001). Internet addiction—reliability and validity in online research. Online-Marktforschung: Beitra ¨ge aus Wissenschaft und Praxis.

[CR29] Hou, Y., Xiong, D., Jiang, T., Song, L., & Wang, Q. (2019). Social media addiction: Its impact, mediation, and intervention. *Cyberpsychology: Journal of psychosocial research on cyberspace, 13*(1).

[CR30] Huang XT, Zhang ZJ (2001). The compiling of adolescence time management disposition inventory. Acta Psychologica Sinica.

[CR50] Instituto Nacional de Estadística (2021). Población que usa Internet (en los últimos tres meses). Tipo de actividades realizadas por Internet. Recuperado de https://www.ine.es/ss/Satellite?L=es_ES&c=INESeccion_C&cid=1259925528782&p=1254735110672&pagename=ProductosYServicios%2FPYSLayout

[CR31] Jenaro C, Flores N, Gómez-Vela M, González-Gil F, Caballo C (2007). Problematic internet andcell-phone use: Psychological, behavioral, and health correlates. Addiction Research & Theory.

[CR32] Kim HS (2001). A comparative study regarding health condition and work stress of nurses working in cancer ward and general ward. Journal of Korean Oncology Nursing.

[CR36] Kormkaz M, Kiran- Esen B (2012). The effects of peer-training about secure internet use on adolescents. Turkish Psychological Counseling and Guidance Journal.

[CR39] Laconi S, Kaliszewska-Czeremska K, Gnisci A, Sergi I, Barke A, Jeromin F, Kuss D (2018). Cross-cultural study of Problematic Internet Use in nine Europeancountries countries. Computers in Human Behavior.

[CR40] Lau, C. (2011). *Internet addiction among university students in China: Risk factors and health outcomes.* Retrieved from https://repository.lib.cuhk.edu.hk/en/item/cuhk-344989

[CR41] Lenhart, A., Smith, A., Anderson, M., Duggan, M., & Perrin, A. (2015). Teens, technology and friendships.

[CR42] Leung L (2017). Stressful life events, motives for internet use, and social support among digital kids. CyberPsychology & Behavior.

[CR43] Li R, Shi G, Ji J, Wang H, Wang W, Li Y, Liu B (2017). A 2-year longitudinal psychological intervention study on the prevention of internet addiction in junior high school students of Jinan city. Biomedical Research.

[CR44] Lin CY, Broström A, Nilsen P, Griffiths MD, Pakpour AH (2017). Psychometric validation of the Persian Bergen Social Media Addiction Scale using classic test theory and Rasch models. Journal of Behavioral Addictions.

[CR45] Lopez-Fernandez O, Kuss DJ (2020). Preventing harmful internet use-related addiction problems in Europe: A literature review and policy options. International Journal of Environmental Research and Public Health.

[CR46] Meerkerk GJ, Van Den Eijnden RJ, Vermulst AA, Garretsen HF (2009). The compulsive internet use scale (CIUS): Some psychometric properties. Cyberpsychology & Behavior.

[CR47] Ministerio de Sanidad, S. S. (n.d.). *Plan de acción sobre adicciones 2018–2020.* Retrieved diciembre 13, 2020, from https://pnsd.sanidad.gob.es/pnsd/planAccion/docs/PLAD_2018-2020_FINAL.pdf

[CR48] Modara, F., Rezaeenour, J., & Sayehmiri, N. (2017, Octubre). Prevalence of Internet Addiction in Iran: A Systematic Review and Meta-analysis. Retrieved from https://www.researchgate.net/publication/329856021_Prevalence_of_Internet_Addiction_in_Iran_A_Systematic_Review_and_Meta-analysis PMC6294487 30574288

[CR87] Moreno, M. A., Jelenchick, L. A., & Christakis, D. A. (2013). Problematic internet use among older adolescents: A conceptual framework. *Computers in Human Behavior*, *29*(4), 1879–1887.

[CR5] National Peer Helpers Association, (2002). Programmatic standards. Retrieved; 01. 05. 2010 from http://www.peerhelping.org

[CR51] Navarro-Mancilla ÁA, Rueda-Jaimes GE (2007). Adicción a Internet: Revisión crítica de la literatura. Revista Colombiana de Psiquiatría.

[CR52] Nicholas LA, Niki R (2004). Development of a psychometrically sound internet addiction scale: A preliminary step. Psychology of Addictive Behaviors.

[CR54] Page MJ, McKenzie JE, Bossuyt PM, Boutron I, Hoffmann TC, Mulrow CD, Moher D (2021). The PRISMA 2020 statement: An updated guideline for reporting systematic reviews. Systematic reviews.

[CR58] Rehbein F, Kleimann M, Mo ¨ßle T (2010). Prevalence and risk factors of video game dependency in adolescence: Results of a German nationwide survey. Cyberpsychology, Behavior, & Social Networking.

[CR59] Rioja, G. D. (2018). *Plan de Prevención de Adicciones de La Rioja 2018–2024.* Retrieved diciembre 13, 2020, from https://www.infodrogas.org/files/Plan-de-Prevencion-de-Adicciones.pdf

[CR60] Sánchez Pardo, L., Benito Corchete, R., Serrano Badía, A., Aleixandre Benavent, R., & Bueno Cañigral, F. J. (2018). *Programa de prevención del uso problemático de internet y redes sociales. “Clickeando”.* Retrieved diciembre 13, 2020, from http://www.codajic.org/sites/www.codajic.org/files/Programa-prevencion-uso-problematico-internet-redes-sociales-Clickeando-Web(1).pdf

[CR61] Sánchez-Carbonell X, Beranuy M, Castellana M, Chamarro A, Oberst U (2008). La adicción a Internet y al móvil: ¿moda o trastorno?. Adicciones.

[CR62] Sanchez-Martinez M, Otero A (2009). Factors associated with cell phone use in adolescentsin the community of Madrid (Spain). CyberPsychology & Behavior.

[CR63] Sánchez-Meca J, Botella J (2010). Revisiones sistemáticas y meta-análisis: Herramientas para la práctica profesional. Papeles del Psicólogo.

[CR64] Secades-Villa R, Calafat A, Fernández-Hermida JR, Juan M, Duch M, Skärstrand E, Talic S (2014). Duration of Internet use and adverse psychosocial effects among European adolescents. Adicciones.

[CR65] Shek DT, Ma CM (2010). Dimensionality of the Chinese positive youth development scale: Confirmatory factor analyses. Social Indicators Research.

[CR66] Shek D, Yu L (2011). Prevention of adolescent problem behavior: longitudinal impact of the project P.A.T.H.S. in Hong Kong. The Scientific World Journal.

[CR71] Tahiroglu AY, Celik G, Uzel M, Ozcan N, Avci A (2008). Internet use among Turkish adolescents. CyberPsychology & Behavior.

[CR72] Throuvala M, Griffiths M, Rennoldson M, Kuss D (2019). School-based prevention for adolescent internet addiction: Prevention is. Current Neuropharmacology.

[CR73] Trahtemberg, L. (2000). El impacto previsible de las nuevas tecnologías en la enseñanza y la organización escolar. *La Revista Iberoamericana de Educación*, 24. Retrieved from https://rieoei.org/historico/documentos/rie24a02.htm

[CR74] Tsitsika AK, Andrie EK, Psaltopoulou T, Tzavara CK, Sergentanis TN, Ntanasis-Stathopoulos I, Tsolia M (2016). Association between problematic internet use, socio-demographic variables and obesity among European adolescents. The European Journal of Public Health.

[CR75] Uysal G, Balci S (2018). Evaluation of a school-based program for internet addiction of adolescents in Turkey. Journal of Addictions Nursing.

[CR76] Vondráčková P, Gabrhelík R (2016). Prevention of Internet addiction: A systematic review. Journal of Behavioral Addictions.

[CR77] Walther B, Hanewinkel R, Morgenstern M (2014). Effects of a brief school-based media literacy interventionon digital media use in adolescents: Cluster randomized controlled trial. Cyberpsychology, Behavior, and Social Networking,.

[CR91] Wang, X. D., Wang, X. L., & Ma, H. (1999). *Mental Health Assessment Scale (revised version)*. Beijing: Chinese Encyclopedia Press.

[CR79] Wu C-Y, Lee M-B, Liao S-C, Ko C-H (2019). A nationwide survey of the prevalence and psychosocial correlates of internet addictive disorders in Taiwan. Journal of the Formosan Medical Association.

[CR81] Yang S-Y, Kim H-S (2018). Effects of a prevention program for internet addiction among middle school students in South Korea. Public Health Nursing.

[CR83] Yılmazhan-Gültutan, Ş. (2007). *İlköğretim okulu 6.,7. ve 8. Sınıf öğrencilerinin internet k u l l a n ı m alışkanlıkları*, Yayınlanmamış Yüksek Lisans Tezi. Ankara Üniversitesi. Eğitim Bilimleri Enstitüsü, Ankara.

[CR85] Yu L, Shek D (2013). Internet addiction in Hong Kong adolescents: A three-year longitudinal study. North American Society for Pediatric and Adolescent Gynecolog.

[CR86] Zan L-L, Liu B-L, Liu Z-X (2008). Development of Internet addiction disorder diagnostic scale for middle school students. Chinese Journal of Clinical Psychology.

