# Supplementary material for: Are School-Based Interventions Effective in Preventing Internet Misuse? A Systematic Review
Source: J Prev (2022). 2023 Dec 7;45(2):193–212. doi: 10.1007/s10935-023-00757-9 (PMC10981624; doi:10.1007/s10935-023-00757-9)
Supplement: Supplementary file 1 — Supplementary file1 (DOCX 151 KB) [file 10935_2023_757_MOESM1_ESM.docx]

***FIGURES***

***Figure 1.***

*Prisma’s* *flowchart of bibliographic search strategy.*

**Identification of studies via other methods**

Results classified after reading the title

**N=137**

References identified through the database search

**N=335**

References identified through the database search

**N=378**

Classified results after reading the abstract

**N=12**

Results classified after reading the text

**N=9**

Excluded for not meeting the inclusion criterio

**N=3**

Excluded for not meeting the inclusion criteria

**N=125**

Excluded by duplicates

**N=43**

Excluded for not meeting the inclusion criteria

**N=198**

Results classified for final analysis

**N=11**

**Identification of studies via databases and registers**

**Included**

**Identification**

**Screening**

Records identified from:

Websites **(N =0)**

Organisations **(N = 0)**

Citation searching **(N = 2)**

etc.

| **TABLES**  **Table 1.**  Date of papers extraction per database | |  |
| --- | --- | --- |
| Database | Results | Date |
| PubMed | 73 | 07/2021 |
| Scopus | 172 | 05/2021 |
| Health Evidence | 47 | 04/2021 |
| Agency for Healthcare Research and Quality | 0 | 04/2021 |
| Guidelines International Network | 0 | 04/2021 |
| ERIC | 15 | 07/2021 |
| QUIEN | 15 | 07/2021 |
| HNT | 0 | 06/2021 |
| Cochrane | 56 | 08/2021 |
| **TOTAL** | **378** |  |

|  | |  | | **Table 2.**  *Description of the selected studies characteristics* | | | | | | | | |
| --- | --- | --- | --- | --- | --- | --- | --- | --- | --- | --- | --- | --- |
| N. | No. | | Author (year) | | Title | Country | Program (Authors) | Design | Sample | B. A. | Quality |  |
| 1 | 1 | | Li et al., (2017) | | A 2-year longitudinal psychological intervention study on the prevention of Internet addiction in junior high school students of Jinan city. | China | Own program: (Li et al.) | Longitudinal 24 months Random: Yes  Cntl.Gr: Yes  Eva. internal | N=759 students.  Gr Exp. n=446. Cntl.Gr. n=385  Hometown: 1 Jinan City High School  Inclusion criteria: no alterations in IADDS and Symptom Checklist 90 SCL-90 | 5 | 22 |  |
| 2 | 2 | | Uysal & Balci, (2018) | | Evaluation of a School-Based Program for Internet Addiction of Adolescents in Turkey. | Turkey | Healthy Internet Use Program  (Uysal & Balci) | Longitudinal 9 months.  Random: No  Cntl.Gr: Yes  Eva. internal | N=64 16–18-year-olds from two high schools.  Gr Exp. n=41, Cntl.Gr. n=43.  Inclusion criteria: > 91 in The Internet Addiction Scale | 4 | 13 |  |
| 3 | 3 | | Yang & Kim, (2018) | | Effects of a prevention program for Internet addiction among middle school students in South Korea. | South Korea | Own program:  (Yang & Kim) | Cross.  Random: No  Cntl.Gr: Yes  Eva. internal | N= 79 8th grade students between 13-15 years old from two secondary schools.  Gr Exp. n=38, Gr. Cntl. n=41.  Inclusion criteria: score more than 108 on Internet Addiction propensity Scale. | 4 | 21 |  |
| 4 | 4 | | Walther, et al. (2014) | | Effects of a Brief School-Based Media Literacy Intervention on Digital Media Use in Adolescents: Cluster Randomized Controlled Trial. | Germany | Vernetzte www.Welten Walther et al. (2014) | Longitudinal 12 months Random: Yes  Cntl.Gr: Yes  Eva. internal | N= 1,843 12-year-olds from 27 schools  Gr Exp. N=922 Gr. Cntl. N=921  Inclusion criteria: have parental permission, be present on the day of data collection. | 3 | 21 |  |
| 5 | 5 | | Kormkaz & Kiran-Esen, (2012) | | The Effects of Peer-Training about Secure Internet Use on Adolescents. | Turkey | Peer Training Program. National Peer Helpers Association, (2002). | Cross.  Random: No  Cntl.Gr: Yes  Eva. external | N= 825 6th, 7th and 8th grade students from primary schools in Mersin (Turkey) between 13 and 15 years old.  exp group _ n=410 Group cont. n=415  Inclusion criteria: have parental permission | 4 | 12 |  |
| 6 | 6 | | Shek & Yu, (2011) | | Prevention of Adolescent Problem  Behaviour: Longitudinal Impact of the  Project PATHS in Hong Kong | Hong Kong | Project PATHS (Positive Adolescent Training through Holistic Social Programs),  Shek & Yu | Longitudinal 36 months. Random: Yes  Cntl.Gr: Yes  Eva. internal | N= 7846 at the beginning of the process from 48 Hong Kong secondary schools.  group count n= 3797 Group exp. n= 4049 | 3 | 23 |  |
| 7 | 7 | | Busch et al. (2013) | | Results of a Multibehavioral Health-Promoting School Pilot Intervention in a Dutch Secondary School | Netherlands | Applied Health Promoting School principles  (Rasmussen & Rivett) | Longitudinal 36 months  Random: No  Cntl.Gr: No  Eva. external | 336 students from 15 to 16 years old (4th grade) | 5 | 11 |  |
| 8 | 8 | | Celik (2016) | | Educational intervention for reducing Internet addiction tendencies | Turkey | Training-Program Sessions to Increase Conscious Internet Use, Academic Motivation, and Efficient Use of  Time. (Celik) | Longitudinal 6 months. Random: Yes  Cntl.Gr: Yes  Eva. internal | N=30 high school students  exp group _ n=15 Group Cont. n=15  Inclusion criteria: students  with scores higher than the mean of the sample in the PIUS | 3 | 18 |  |
| 9 | 9 | | de Leeuw et al. (2010). | | Internet and game behaviour at a secondary school and a newly developed health promotion programme: A prospective study | countries Low | The media literacy of children and young people. Buckingham (2005) | Longitudinal 12 months  Random: No  Cntl.Gr: No  Eva. external | 475 high school students aged 11-18. That they were present on the days of carrying out the pre- and post-treatment tests | 4 | 16 |  |
| 10 | 10 | | Du et al. (2010) | | Longer term effect of randomized, controlled group cognitive behavioural therapy for Internet addiction in adolescent students in Shanghai | Shanghai | Cognitive behavioural therapy group | Longitudinal 6 months Random: Yes  Cntl.Gr: Yes  Eva. external | 56 students with internet addiction according to Beard and Wolf criteria, with a mean of 16.63 ± 1.23 years  exp group _ n=32 Group Cont. n=24 | 5 | 19 |  |
| 11 | 11 | | Hou et al. (2019) | | Social media addiction: Its impact, mediation, and intervention | China | Ad hoc intervention program with cognitive restructuring for addiction to social networks based on the work of Young's | Cross  Random: Yes  Cntl.Gr: Yes  Eva. internal | 38 students from Peking University with a mean age of 19.71 ± 1.43 years.  Exp.Gr n=18 Cnt.Gr n=20 | 5 | 10 |  |
|  |  | | *B. A.= Bias Assessment | | | | | | | | |  |

|  | **Table 3.**  *Description of the selected studies characteristics and results*   \| *n*o. \| Program \| Goals \| Results \| Variable \| Instrument \| \| --- \| --- \| --- \| --- \| --- \| --- \| \| 1 \| N. Sessions: 5 in each block  Frequency: 1 block at the beginning of each semester  Duration: 1h  Facilitator: psychiatrists and teachers  Techniques: psychoeducation \| -Improve mental health  -Prevent internet addiction \| ⇑Mental health  ⇓Internet addiction \| IA Severity  General symptoms \| IADDS (Zan, 2008)  SCL-90 (Wang, Wang & Ma, 1999) \| \| 2 \| N. Sessions: 8  Frequency: no data  Duration: between 40 and 80 min  Facilitator: psychologist  Techniques: watch movies, play games, summarize, draw, make posters, organize and use flashcards \| - Reduce the use of the Internet  - Develop healthy use habits  - Learn to better organize daily life activities  - Raise parents' awareness of healthy Internet use by children \| ⇓Internet addiction compared with the measurements made at 3 and 9 months later. \| IA Severity \| IAS (Nicholas & Niki, 2004)  Interview with parents  Weekly calls to parents \| \| 3 \| N. Sessions: 10  Weekly frequency  Duration: 45 min/ week  Facilitator: School Nurse  Techniques: psychoeducation, role playing, group activities and written tasks. \| -Work on self-efficacy and self-regulation.  -Study the effect of these skills on Internet use time, Internet addiction and self-control. \| ⇑Self-regulation and self-efficacy  - Internet usage time  Exp.Gr < Cntl.Gr  - Internet addiction  Exp.Gr < Cntl.Gr  Self-regulation, self-efficacy and self-control predictors of problematic Internet use \| Self-control  Self-efficacy  IA Severity \| Brief-Self Control Scale (Cho, 2011)  Self-Efficacy Scale (Kim, 2001)  K-Scale. Internet Addiction Prone Scale (Agency, 2003) \| \| 4 \| N. Sessions: 4  Weekly frequency  Duration: 90 min/ session  Facilitator: trained teacher  Techniques: psychoeducation, self-observation, debate and student reflection \| -Work on students' self-reflection and self-control to reduce the problematic use of the Internet and reduce computer games. \| ⇓Time spent on games.  Internet abuse and addiction  Exp.Gr = Cntl.Gr \| Severity addiction video game  IA \| KFN-CSAS-II (Rehbein, Kleimann, & Mößle, 2010)  ISS (Hahn & Jerusalem, 2001)  Observation of parents and house rules  Data sociodemographic \| \| 5 \| N. Sessions: 10  Weekly frequency  Duration: 90 minutes  Dynamizer: prepared adolescents  Techniques: psychoeducation and group activities. \| - Work on skills related to the Internet through peer training. \| Internet usage time  Exp.Gr < G. Cntl  Quality of Internet use. Exp.Gr > G. Cntl. \| Internet usage habits \| The Internet Use Habit Scale (Yılmazhan-Gültutan, 2007) \| \| 6 \| N. Sessions: 40 per course  Frequency: bi-weekly  Duration: at least 30 min/ session for 3 courses  Dynamizer: prepared professionals  Techniques: holistic social programs \| - To study the longitudinal effects of the PATHS program in the prevention of problem behaviours in young people. \| control and self-control  Exp.Gr > G. Cntl.  Internet addiction and abuse  Exp.Gr < G. Cntl. \| Self-monitoring on the internet  Positive Youth Development Measure \| Internet Use Control  Chinese Positive  Youth Development Scale (CPYDS) (Shek & Ma, 2010) \| \| 7 \| N. Sessions: 30 per course  Weekly frequency  Duration: 50 minutes  Facilitator: trained teacher  Techniques: psychoeducation and group activities. \| - Give tools to students  enable them to make healthy choices  - Reduce excessive compulsive behaviour related to internet use and gaming \| Compulsive internet use  ⇓Women  = Males  video game addiction  No significant changes \| Health survey  Socioeconomic status  Prosocial behaviour  Compulsive internet use  Video game addiction \| Dutch Health Behaviour in School-aged Children (HBSC)  The Family Affluence Scale (FAS) (Boyce, Torsheim, Currie & Zambon, 2006)  Strengths and Difficulties Questionnaire (SDQ) (Goodman, Meltzer & Bailey, 1998)  Compulsive Internet Use Scale (CIUS) (Meerkerk, Van Den Eijnden, Vermulst & Garretsen, 2009)  Videogame Addiction Test (VAT) (Derived from CIUS) \| \| 8 \| N. Sessions: 5  Weekly frequency  Duration: from 90 to 120 min  Facilitator: trained teacher  Techniques: psychoeducation \| - Reduce the time that study group members spent on the Internet  - Improve awareness by increasing your academic motivation  - Reduce internet addiction \| Internet addiction  Exp.Gr < G. Cntl.  academic motivation  Exp.Gr > G. Cntl. \| Problematic internet use \| The Problematic Internet Use Scale (PIUS) (Ceyhan, Ceyhan,  and Gurcan, 2007) \| \| 9 \| N. Sessions: 64  Frequency: bi-weekly  Duration: 1h  Facilitator: trained teacher  Techniques: psychoeducation \| - Prevent internet addiction and online games \| ⇓Online gaming users  ⇓Heavy internet use \| Compulsive internet use  Compulsive use of online gambling  prosocial behaviour  Socioeconomic status \| The Compulsive Internet Use Scale  (CIUS) (Meerkerk et al., 2009)  Compulsive Game Use Scale (CGUS) (Adaptation of CIUS)  Strengths and Difficulties Questionnaire (SDQ) (Goodman, Meltzer & Bailey, 1998)  Family Affluence Scale (FAS) (Boyce, Torsheim, Currie & Zambon, 2006) \| \| 10 \| N. Sessions: 8  Weekly frequency  Duration: between 1.5h and 2h  Facilitator: 2 psychiatrists  Techniques: Cognitive behavioural group therapy and psychoeducation \| - Reduce internet usage  - Increase time management skills, emotional and cognitive management  - Improve emotional state \| ⇓Internet use  Exp.Gr = Cntl.Gr  Time management skills, emotional management  Gr. Exp > G. Cntl  Improved emotional state and self-management  Exp.Gr > Cntl.Gr _ \| IA  Severity of abusive internet use  Perceived control of free time  prosocial behaviour \| Beard's Diagnostic Questionnaire for Internet addiction (Du, Kou & Coghill, 2008)  Internet Overuse Self-Rating Scale (Cao, Su & Gao, 2006)  The Time Management Disposition Scale (TMDS) (Huang & Zhang, 2001)  Strength and Difficulties Questionnaire (Chinese edition) (Goodman, Meltzer & Bailey, 1998) \| \| 11 \| N. Sessions: 4  Weekly frequency  Duration: 1 hour  Facilitator: psychologist  Techniques: Cognitive restructuring \| - Reduce addiction to social networks  - Improve the mental health of students  - Increase your academic efficiency \| Internet and social media addiction  Exp.Gr < Cntl.Gr  ⇑Mental health and academic efficiency \| social media addiction \| Bergen Social Media Addiction Scale (BSMAS) (Lin, Broström, Nilsen, Griffiths & Pakpour, 2017)  Ad hoc questionnaire on hours of sleep, hours of internet use \|   ⇑= Increases; ⇓decreases; = There are no differences  Exp.Gr. = Experimental Group Cntl.Gr = Control Group |
| --- | --- | --- | --- | --- | --- | --- | --- | --- | --- | --- | --- | --- | --- | --- | --- | --- | --- | --- | --- | --- | --- | --- | --- | --- | --- | --- | --- | --- | --- | --- | --- | --- | --- | --- | --- | --- | --- | --- | --- | --- | --- | --- | --- | --- | --- | --- | --- | --- | --- | --- | --- | --- | --- | --- | --- | --- | --- | --- | --- | --- | --- | --- | --- | --- | --- | --- | --- | --- | --- | --- | --- | --- | --- |

**Appendix 1.** *PRISMA checklist.*

| **Section and Topic** | **Item #** | **Checklist item** | **Location where item is reported** |
| --- | --- | --- | --- |
| **TITLE** | | |  |
| Title | 1 | Identify the report as a systematic review. | p.1 Title |
| **ABSTRACT** | | |  |
| Abstract | 2 | See the PRISMA 2020 for Abstracts checklist. | p.1 Abstract |
| **INTRODUCTION** | | |  |
| Rationale | 3 | Describe the rationale for the review in the context of existing knowledge. | pp.2-5 Introduction |
| Objectives | 4 | Provide an explicit statement of the objective(s) or question(s) the review addresses. | p.5 Introduction |
| **METHODS** | | |  |
| Eligibility criteria | 5 | Specify the inclusion and exclusion criteria for the review and how studies were grouped for the syntheses. | p.6 Methods |
| Information sources | 6 | Specify all databases, registers, websites, organisations, reference lists and other sources searched or consulted to identify studies. Specify the date when each source was last searched or consulted. | Table 1 |
| Search strategy | 7 | Present the full search strategies for all databases, registers and websites, including any filters and limits used. | p.6 Methods |
| Selection process | 8 | Specify the methods used to decide whether a study met the inclusion criteria of the review, including how many reviewers screened each record and each report retrieved, whether they worked independently, and if applicable, details of automation tools used in the process. | pp.6-7 Methods |
| Data collection process | 9 | Specify the methods used to collect data from reports, including how many reviewers collected data from each report, whether they worked independently, any processes for obtaining or confirming data from study investigators, and if applicable, details of automation tools used in the process. | pp. 6-7 Methods and Figure1 |
| Data items | 10a | List and define all outcomes for which data were sought. Specify whether all results that were compatible with each outcome domain in each study were sought (e.g., for all measures, time points, analyses), and if not, the methods used to decide which results to collect. | pp.6-7 Methods |
|  | 10b | List and define all other variables for which data were sought (e.g., participant and intervention characteristics, funding sources). Describe any assumptions made about any missing or unclear information. | pp. 6-7 Methods |
| Study risk of bias assessment | 11 | Specify the methods used to assess risk of bias in the included studies, including details of the tool(s) used, how many reviewers assessed each study and whether they worked independently, and if applicable, details of automation tools used in the process. | pp. 6-7 Methods |
| Effect measures | 12 | Specify for each outcome the effect measure(s) (e.g., risk ratio, mean difference) used in the synthesis or presentation of results. | Appendix 2 |
| Synthesis methods | 13a | Describe the processes used to decide which studies were eligible for each synthesis (e.g., tabulating the study intervention characteristics and comparing against the planned groups for each synthesis (item #5)). | pp.6-7 Methods |
|  | 13b | Describe any methods required to prepare the data for presentation or synthesis, such as handling of missing summary statistics, or data conversions. | - |
|  | 13c | Describe any methods used to tabulate or visually display results of individual studies and syntheses. | Figure 1. Tables 1, 2 & 3 |
|  | 13d | Describe any methods used to synthesize results and provide a rationale for the choice(s). If meta-analysis was performed, describe the model(s), method(s) to identify the presence and extent of statistical heterogeneity, and software package(s) used. | Figure 1. Tables 1, 2 & 3 |
|  | 13e | Describe any methods used to explore possible causes of heterogeneity among study results (e.g., subgroup analysis, meta-regression). | Table 3 |
|  | 13f | Describe any sensitivity analyses conducted to assess robustness of the synthesized results. | Table 3 |
| Reporting bias assessment | 14 | Describe any methods used to assess risk of bias due to missing results in a synthesis (arising from reporting biases). | Tables 2 & 3 |
| Certainty assessment | 15 | Describe any methods used to assess certainty (or confidence) in the body of evidence for an outcome. | Limitations |
| **RESULTS** | | |  |
| Study selection | 16a | Describe the results of the search and selection process, from the number of records identified in the search to the number of studies included in the review, ideally using a flow diagram. | Figure 1 |
|  | 16b | Cite studies that might appear to meet the inclusion criteria, but which were excluded, and explain why they were excluded. | pp.6-7 Methods and Table 2 |
| Study characteristics | 17 | Cite each included study and present its characteristics. | Tables 2 & 3 |
| Risk of bias in studies | 18 | Present assessments of risk of bias for each included study. | Tables 2 & 3 |
| Results of individual studies | 19 | For all outcomes, present, for each study: (a) summary statistics for each group (where appropriate) and (b) an effect estimates and its precision (e.g., confidence/credible interval), ideally using structured tables or plots. | Tables 2 & 3 |
| Results of syntheses | 20a | For each synthesis, briefly summarise the characteristics and risk of bias among contributing studies. | Results |
|  | 20b | Present results of all statistical syntheses conducted. If meta-analysis was done, present for each the summary estimate and its precision (e.g., confidence/credible interval) and measures of statistical heterogeneity. If comparing groups, describe the direction of the effect. | - |
|  | 20c | Present results of all investigations of possible causes of heterogeneity among study results. | Table 3 |
|  | 20d | Present results of all sensitivity analyses conducted to assess the robustness of the synthesized results. | - |
| Reporting biases | 21 | Present assessments of risk of bias due to missing results (arising from reporting biases) for each synthesis assessed. | Results |
| Certainty of evidence | 22 | Present assessments of certainty (or confidence) in the body of evidence for each outcome assessed. | Results |
| **DISCUSSION** | | |  |
| Discussion | 23a | Provide a general interpretation of the results in the context of other evidence. | pp.11-15 Discussion |
|  | 23b | Discuss any limitations of the evidence included in the review. | Discussion |
|  | 23c | Discuss any limitations of the review processes used. | Limitations |
|  | 23d | Discuss implications of the results for practice, policy, and future research. | Conclusions |
| **OTHER INFORMATION** | | |  |
| Registration and protocol | 24a | Provide registration information for the review, including register name and registration number, or state that the review was not registered. | - |
|  | 24b | Indicate where the review protocol can be accessed, or state that a protocol was not prepared. | Table 1 |
|  | 24c | Describe and explain any amendments to information provided at registration or in the protocol. | - |
| Support | 25 | Describe sources of financial or non-financial support for the review, and the role of the funders or sponsors in the review. | - |
| Competing interests | 26 | Declare any competing interests of review authors. | Title Page |
| Availability of data, code and other materials | 27 | Report which of the following are publicly available and where they can be found template data collection forms; data extracted from included studies; data used for all analyses; analytic code; any other materials used in the review. | Table 1 |

**Appendix 2.** *CONSORT 2010 quality checklist.*

**Li et al. 2017**


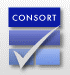
**CONSORT 2010** checklist of information to include when reporting a randomized clinical trial *

| Section/topic | Item No. | Checklist item | Point |
| --- | --- | --- | --- |
| **Title and abstract** | | |  |
|  | 1a | Identified as a randomized trial in the title | 1 |
|  | 1b | Structured summary of trial design, methods, results and conclusions (for specific guidance, see "CONSORT for abstracts") | 1 |
| **Introduction** | | |  |
| Background and objectives | 2a | Scientific background and justification | 1 |
|  | 2b | Specific objectives or hypotheses | 1 |
| **Methods** |  |  |  |
| Test design | 3a | Description of the assay design (e.g., parallel, factorial), including allocation ratio | 1 |
|  | 3b | Important changes in methods after trial initiation (e.g., selection criteria) and their justification | 1 |
| Participants | 4a | Participant selection criteria | 1 |
|  | 4b | Origin (centers and institutions) where the data were recorded | 1 |
| Interventions | 5 | Interventions for each group with sufficient detail to allow replication, including how and when they were actually delivered | 1 |
| Results | 6a | A priori specification of primary and secondary response (or outcome) variables, including how and when they were assessed | 1 |
|  | 6b | Any changes in response variables after trial initiation, together with reasons for modification(s) | 1 |
| Sample size | 7a | How the sample size was determined |  |
|  | 7b | If applicable, explain any interim analyses and interruption rules |  |
| Randomization: | | |  |
| Generation of sequenccia | 8a | Method used to generate the random assignment sequence |  |
|  | 8b | Type of randomization; Details of any restrictions (such as blocks and block sizes) |  |
| Allocation concealment mechanism | 9 | Mechanism used to implement the random assignment sequence (such as sequentially numbered containers), describing the steps taken to hide the sequence until the interventions were assigned |  |
| Implementation | 10 | Who generated the random allocation sequence, who selected participants, and who allocated participants to interventions |  |
| Masking | 11a | If it did, who was blinded after the interventions were allocated (e.g., participants, caregivers, outcome assessors) and how |  |
|  | 11b | If relevant, description of similarity of interventions |  |

| Statistical methods | 12a | Statistical methods used to compare groups in terms of primary and secondary response variables | 1 |
| --- | --- | --- | --- |
|  | 12b | Additional analysis methods, such as subgroup analysis and adjusted analyses | 1 |
| **Results** | | |  |
| Participant flow (a flowchart is strongly recommended) | 13a | For each group, the number of participants who were randomly assigned, who received the proposed treatment, and who were included in the main analysis | 1 |
|  | 13b | For each group, losses and exclusions after randomization, together with the reasons | 1 |
| Recruitment | 14a | Dates defining recruitment and follow-up periods |  |
|  | 14b | Cause of termination or interruption of the trial |  |
| Baseline data | 15 | A table showing baseline demographic and clinical characteristics for each group |  |
| Analyzed numbers | 16 | For each group, number of participants (denominator) included in each analysis and whether the analysis was based on the initially assigned groups | 1 |
| Results and estimation | 17a | For each primary and secondary endpoint or outcome, the results for each group, the estimated effect size, and its precision (as 95% confidence interval) | 1 |
|  | 17b | For dichotomous responses, we recommend the presentation of both absolute and relative effect sizes |  |
| Secondary scans | 18 | Results of any other analyses performed, including subgroup analysis and adjusted analyses, differentiating between those specified a priori and exploratory analyses | 1 |
| Damages | 19 | All unintended harm or effect in each group (for specific guidance, see "CONSORT for harms") |  |
| **Discussion** | | |  |
| Limitations | 20 | Limitations of the study, addressing sources of possible bias, imprecision and, where appropriate, multiplicity of analyses | 1 |
| Generalization | 21 | Possibility of generalization (external validity, applicability) of trial findings | 1 |
| Interpretation | 22 | Interpretation consistent with the results, with balance of benefits and harms, and considering other relevant evidence | 1 |
| **Other information** | | |  |
| Registration | 23 | Registration number and name of the trial register |  |
| Protocol | 24 | Where the full assay protocol can be accessed, if available |  |
| Financing | 25 | Sources of funding and other support (such as supply of medicines), role of funders | 1 |

* We strongly recommend reading this checklist together with "the CONSORT 2010 Explanation and Elaboration" to clarify important questions about all items. If applicable, we also recommend reading CONSORT extensions for cluster-randomized trials, non-inferiority and equivalence trials, non-pharmacological treatments, herbal medicine interventions, and pragmatic trials. Other extensions are being prepared: for these and for relevant updated references, related to this checklist, see [www.consort-statement.org](http://www.consort-statement.org)

**22**

**Uysal & Balci, 2018**


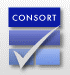
**CONSORT 2010** checklist of information to include when reporting a randomized clinical trial *

| Section/topic | Item No. | Checklist item | Point |
| --- | --- | --- | --- |
| **Title and abstract** | | |  |
|  | 1a | Identified as a randomized trial in the title |  |
|  | 1b | Structured summary of trial design, methods, results and conclusions (for specific guidance, see "CONSORT for abstracts") |  |
| **Introduction** | | |  |
| Background and objectives | 2a | Scientific background and justification | 1 |
|  | 2b | Specific objectives or hypotheses |  |
| **Methods** |  |  |  |
| Test design | 3a | Description of the assay design (e.g., parallel, factorial), including allocation ratio |  |
|  | 3b | Important changes in methods after trial initiation (e.g., selection criteria) and their justification |  |
| Participants | 4a | Participant selection criteria | 1 |
|  | 4b | Origin (centers and institutions) where the data were recorded |  |
| Interventions | 5 | Interventions for each group with sufficient detail to allow replication, including how and when they were actually delivered | 1 |
| Results | 6a | A priori specification of primary and secondary response (or outcome) variables, including how and when they were assessed |  |
|  | 6b | Any changes in response variables after trial initiation, together with reasons for modification(s) |  |
| Sample size | 7a | How the sample size was determined | 1 |
|  | 7b | If applicable, explain any interim analyses and interruption rules |  |
| Randomization: | | |  |
| Generation of sequenccia | 8a | Method used to generate the random assignment sequence | 1 |
|  | 8b | Type of randomization; Details of any restrictions (such as blocks and block sizes) | 1 |
| Allocation concealment mechanism | 9 | Mechanism used to implement the random assignment sequence (such as sequentially numbered containers), describing the steps taken to hide the sequence until the interventions were assigned |  |
| Implementation | 10 | Who generated the random allocation sequence, who selected participants, and who allocated participants to interventions |  |
| Masking | 11a | If it did, who was blinded after the interventions were allocated (e.g., participants, caregivers, outcome assessors) and how |  |
|  | 11b | If relevant, description of similarity of interventions |  |

| Statistical methods | 12a | Statistical methods used to compare groups in terms of primary and secondary response variables | 1 |
| --- | --- | --- | --- |
|  | 12b | Additional analysis methods, such as subgroup analysis and adjusted analyses |  |
| **Results** | | |  |
| Participant flow (a flowchart is strongly recommended) | 13a | For each group, the number of participants who were randomly assigned, who received the proposed treatment, and who were included in the main analysis |  |
|  | 13b | For each group, losses and exclusions after randomization, together with the reasons | 1 |
| Recruitment | 14a | Dates defining recruitment and follow-up periods |  |
|  | 14b | Cause of termination or interruption of the trial |  |
| Baseline data | 15 | A table showing baseline demographic and clinical characteristics for each group | 1 |
| Analyzed numbers | 16 | For each group, number of participants (denominator) included in each analysis and whether the analysis was based on the initially assigned groups | 1 |
| Results and estimation | 17a | For each primary and secondary endpoint or outcome, the results for each group, the estimated effect size, and its precision (as 95% confidence interval) |  |
|  | 17b | For dichotomous responses, we recommend the presentation of both absolute and relative effect sizes |  |
| Secondary scans | 18 | Results of any other analyses performed, including subgroup analysis and adjusted analyses, differentiating between those specified a priori and exploratory analyses |  |
| Damages | 19 | All unintended harm or effect in each group (for specific guidance, see "CONSORT for harms") |  |
| **Discussion** | | |  |
| Limitations | 20 | Limitations of the study, addressing sources of possible bias, imprecision and, where appropriate, multiplicity of analyses | 1 |
| Generalization | 21 | Possibility of generalization (external validity, applicability) of trial findings | 1 |
| Interpretation | 22 | Interpretation consistent with the results, with balance of benefits and harms, and considering other relevant evidence | 1 |
| **Other information** | | |  |
| Registration | 23 | Registration number and name of the trial register |  |
| Protocol | 24 | Where the full assay protocol can be accessed, if available |  |
| Financing | 25 | Sources of funding and other support (such as supply of medicines), role of funders |  |

* We strongly recommend reading this checklist together with "the CONSORT 2010 Explanation and Elaboration" to clarify important questions about all items. If applicable, we also recommend reading CONSORT extensions for cluster-randomized trials, non-inferiority and equivalence trials, non-pharmacological treatments, herbal medicine interventions, and pragmatic trials. Other extensions are being prepared: for these and for relevant updated references, related to this checklist, see [www.consort-statement.org](http://www.consort-statement.org)

**13**

**Yang & Kim, 2018**


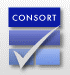
**CONSORT 2010** checklist of information to include when reporting a randomized clinical trial *

| Section/topic | Item No. | Checklist item | Point |
| --- | --- | --- | --- |
| **Title and abstract** | | |  |
|  | 1a | Identified as a randomized trial in the title |  |
|  | 1b | Structured summary of trial design, methods, results and conclusions (for specific guidance, see "CONSORT for abstracts") | 1 |
| **Introduction** | | |  |
| Background and objectives | 2a | Scientific background and justification | 1 |
|  | 2b | Specific objectives or hypotheses | 1 |
| **Methods** |  |  | 1 |
| Test design | 3a | Description of the assay design (e.g., parallel, factorial), including allocation ratio | 1 |
|  | 3b | Important changes in methods after trial initiation (e.g., selection criteria) and their justification |  |
| Participants | 4a | Participant selection criteria | 1 |
|  | 4b | Origin (centers and institutions) where the data were recorded |  |
| Interventions | 5 | Interventions for each group with sufficient detail to allow replication, including how and when they were actually delivered | 1 |
| Results | 6a | A priori specification of primary and secondary response (or outcome) variables, including how and when they were assessed | 1 |
|  | 6b | Any changes in response variables after trial initiation, together with reasons for modification(s) |  |
| Sample size | 7a | How the sample size was determined | 1 |
|  | 7b | If applicable, explain any interim analyses and interruption rules |  |
| Randomization: | | |  |
| Generation of sequenccia | 8a | Method used to generate the random assignment sequence |  |
|  | 8b | Type of randomization; Details of any restrictions (such as blocks and block sizes) |  |
| Allocation concealment mechanism | 9 | Mechanism used to implement the random assignment sequence (such as sequentially numbered containers), describing the steps taken to hide the sequence until the interventions were assigned |  |
| Implementation | 10 | Who generated the random allocation sequence, who selected participants, and who allocated participants to interventions |  |
| Masking | 11a | If it did, who was blinded after the interventions were allocated (e.g., participants, caregivers, outcome assessors) and how |  |
|  | 11b | If relevant, description of similarity of interventions |  |

| Statistical methods | 12a | Statistical methods used to compare groups in terms of primary and secondary response variables | 1 |
| --- | --- | --- | --- |
|  | 12b | Additional analysis methods, such as subgroup analysis and adjusted analyses | 1 |
| **Results** | | |  |
| Participant flow (a flowchart is strongly recommended) | 13a | For each group, the number of participants who were randomly assigned, who received the proposed treatment, and who were included in the main analysis | 1 |
|  | 13b | For each group, losses and exclusions after randomization, together with the reasons | 1 |
| Recruitment | 14a | Dates defining recruitment and follow-up periods |  |
|  | 14b | Cause of termination or interruption of the trial |  |
| Baseline data | 15 | A table showing baseline demographic and clinical characteristics for each group | 1 |
| Analyzed numbers | 16 | For each group, number of participants (denominator) included in each analysis and whether the analysis was based on the initially assigned groups | 1 |
| Results and estimation | 17a | For each primary and secondary endpoint or outcome, the results for each group, the estimated effect size, and its precision (as 95% confidence interval) | 1 |
|  | 17b | For dichotomous responses, we recommend the presentation of both absolute and relative effect sizes |  |
| Secondary scans | 18 | Results of any other analyses performed, including subgroup analysis and adjusted analyses, differentiating between those specified a priori and exploratory analyses | 1 |
| Damages | 19 | All unintended harm or effect in each group (for specific guidance, see "CONSORT for harms") |  |
| **Discussion** | | |  |
| Limitations | 20 | Limitations of the study, addressing sources of possible bias, imprecision and, where appropriate, multiplicity of analyses | 1 |
| Generalization | 21 | Possibility of generalization (external validity, applicability) of trial findings | 1 |
| Interpretation | 22 | Interpretation consistent with the results, with balance of benefits and harms, and considering other relevant evidence | 1 |
| **Other information** | | |  |
| Registration | 23 | Registration number and name of the trial register |  |
| Protocol | 24 | Where the full assay protocol can be accessed, if available | 1 |
| Financing | 25 | Sources of funding and other support (such as supply of medicines), role of funders |  |

* We strongly recommend reading this checklist together with "the CONSORT 2010 Explanation and Elaboration" to clarify important questions about all items. If applicable, we also recommend reading CONSORT extensions for cluster-randomized trials, non-inferiority and equivalence trials, non-pharmacological treatments, herbal medicine interventions, and pragmatic trials. Other extensions are being prepared: for these and for relevant updated references, related to this checklist, see [www.consort-statement.org](http://www.consort-statement.org)

**21**

**Walther, Hanewinkel, & Morgenstern, 2014**


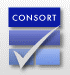
**CONSORT 2010** checklist of information to include when reporting a randomized clinical trial *

| Section/topic | Item No. | Checklist item | Point |
| --- | --- | --- | --- |
| **Title and abstract** | | |  |
|  | 1a | Identified as a randomized trial in the title | 1 |
|  | 1b | Structured summary of trial design, methods, results and conclusions (for specific guidance, see "CONSORT for abstracts") |  |
| **Introduction** | | |  |
| Background and objectives | 2a | Scientific background and justification | 1 |
|  | 2b | Specific objectives or hypotheses |  |
| **Methods** |  |  |  |
| Test design | 3a | Description of the assay design (e.g., parallel, factorial), including allocation ratio | 1 |
|  | 3b | Important changes in methods after trial initiation (e.g., selection criteria) and their justification |  |
| Participants | 4a | Participant selection criteria | 1 |
|  | 4b | Origin (centers and institutions) where the data were recorded |  |
| Interventions | 5 | Interventions for each group with sufficient detail to allow replication, including how and when they were actually delivered | 1 |
| Results | 6a | A priori specification of primary and secondary response (or outcome) variables, including how and when they were assessed | 1 |
|  | 6b | Any changes in response variables after trial initiation, together with reasons for modification(s) |  |
| Sample size | 7a | How the sample size was determined |  |
|  | 7b | If applicable, explain any interim analyses and interruption rules |  |
| Randomization: | | |  |
| Generation of sequenccia | 8a | Method used to generate the random assignment sequence | 1 |
|  | 8b | Type of randomization; Details of any restrictions (such as blocks and block sizes) |  |
| Allocation concealment mechanism | 9 | Mechanism used to implement the random assignment sequence (such as sequentially numbered containers), describing the steps taken to hide the sequence until the interventions were assigned | 1 |
| Implementation | 10 | Who generated the random allocation sequence, who selected participants, and who allocated participants to interventions | 1 |
| Masking | 11a | If it did, who was blinded after the interventions were allocated (e.g., participants, caregivers, outcome assessors) and how |  |
|  | 11b | If relevant, description of similarity of interventions |  |

| Statistical methods | 12a | Statistical methods used to compare groups in terms of primary and secondary response variables | 1 |
| --- | --- | --- | --- |
|  | 12b | Additional analysis methods, such as subgroup analysis and adjusted analyses |  |
| **Results** | | |  |
| Participant flow (a flowchart is strongly recommended) | 13a | For each group, the number of participants who were randomly assigned, who received the proposed treatment, and who were included in the main analysis | 1 |
|  | 13b | For each group, losses and exclusions after randomization, together with the reasons | 1 |
| Recruitment | 14a | Dates defining recruitment and follow-up periods |  |
|  | 14b | Cause of termination or interruption of the trial |  |
| Baseline data | 15 | A table showing baseline demographic and clinical characteristics for each group | 1 |
| Analyzed numbers | 16 | For each group, number of participants (denominator) included in each analysis and whether the analysis was based on the initially assigned groups | 1 |
| Results and estimation | 17a | For each primary and secondary endpoint or outcome, the results for each group, the estimated effect size, and its precision (as 95% confidence interval) | 1 |
|  | 17b | For dichotomous responses, we recommend the presentation of both absolute and relative effect sizes |  |
| Secondary scans | 18 | Results of any other analyses performed, including subgroup analysis and adjusted analyses, differentiating between those specified a priori and exploratory analyses | 1 |
| Damages | 19 | All unintended harm or effect in each group (for specific guidance, see "CONSORT for harms") |  |
| **Discussion** | | |  |
| Limitations | 20 | Limitations of the study, addressing sources of possible bias, imprecision and, where appropriate, multiplicity of analyses | 1 |
| Generalization | 21 | Possibility of generalization (external validity, applicability) of trial findings | 1 |
| Interpretation | 22 | Interpretation consistent with the results, with balance of benefits and harms, and considering other relevant evidence | 1 |
| **Other information** | | |  |
| Registration | 23 | Registration number and name of the trial register |  |
| Protocol | 24 | Where the full assay protocol can be accessed, if available | 1 |
| Financing | 25 | Sources of funding and other support (such as supply of medicines), role of funders | 1 |

* We strongly recommend reading this checklist together with "the CONSORT 2010 Explanation and Elaboration" to clarify important questions about all items. If applicable, we also recommend reading CONSORT extensions for cluster-randomized trials, non-inferiority and equivalence trials, non-pharmacological treatments, herbal medicine interventions, and pragmatic trials. Other extensions are being prepared: for these and for relevant updated references, related to this checklist, see [www.consort-statement.org](http://www.consort-statement.org)

**21**

**Kormkaz & Kiran- Esen, 2012**


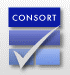
**CONSORT 2010** checklist of information to include when reporting a randomized clinical trial *

| Section/topic | Item No. | Checklist item | Point |
| --- | --- | --- | --- |
| **Title and abstract** | | |  |
|  | 1a | Identified as a randomized trial in the title |  |
|  | 1b | Structured summary of trial design, methods, results and conclusions (for specific guidance, see "CONSORT for abstracts") |  |
| **Introduction** | | |  |
| Background and objectives | 2a | Scientific background and justification | 1 |
|  | 2b | Specific objectives or hypotheses |  |
| **Methods** |  |  |  |
| Test design | 3a | Description of the assay design (e.g., parallel, factorial), including allocation ratio | 1 |
|  | 3b | Important changes in methods after trial initiation (e.g., selection criteria) and their justification |  |
| Participants | 4a | Participant selection criteria |  |
|  | 4b | Origin (centers and institutions) where the data were recorded |  |
| Interventions | 5 | Interventions for each group with sufficient detail to allow replication, including how and when they were actually delivered |  |
| Results | 6a | A priori specification of primary and secondary response (or outcome) variables, including how and when they were assessed | 1 |
|  | 6b | Any changes in response variables after trial initiation, together with reasons for modification(s) |  |
| Sample size | 7a | How the sample size was determined |  |
|  | 7b | If applicable, explain any interim analyses and interruption rules |  |
| Randomization: | | |  |
| Generation of sequenccia | 8a | Method used to generate the random assignment sequence |  |
|  | 8b | Type of randomization; Details of any restrictions (such as blocks and block sizes) |  |
| Allocation concealment mechanism | 9 | Mechanism used to implement the random assignment sequence (such as sequentially numbered containers), describing the steps taken to hide the sequence until the interventions were assigned |  |
| Implementation | 10 | Who generated the random allocation sequence, who selected participants, and who allocated participants to interventions |  |
| Masking | 11a | If it did, who was blinded after the interventions were allocated (e.g., participants, caregivers, outcome assessors) and how |  |
|  | 11b | If relevant, description of similarity of interventions |  |

| Statistical methods | 12a | Statistical methods used to compare groups in terms of primary and secondary response variables | 1 |
| --- | --- | --- | --- |
|  | 12b | Additional analysis methods, such as subgroup analysis and adjusted analyses | 1 |
| **Results** | | |  |
| Participant flow (a flowchart is strongly recommended) | 13a | For each group, the number of participants who were randomly assigned, who received the proposed treatment, and who were included in the main analysis | 1 |
|  | 13b | For each group, losses and exclusions after randomization, together with the reasons |  |
| Recruitment | 14a | Dates defining recruitment and follow-up periods |  |
|  | 14b | Cause of termination or interruption of the trial |  |
| Baseline data | 15 | A table showing baseline demographic and clinical characteristics for each group | 1 |
| Analyzed numbers | 16 | For each group, number of participants (denominator) included in each analysis and whether the analysis was based on the initially assigned groups | 1 |
| Results and estimation | 17a | For each primary and secondary endpoint or outcome, the results for each group, the estimated effect size, and its precision (as 95% confidence interval) | 1 |
|  | 17b | For dichotomous responses, we recommend the presentation of both absolute and relative effect sizes |  |
| Secondary scans | 18 | Results of any other analyses performed, including subgroup analysis and adjusted analyses, differentiating between those specified a priori and exploratory analyses |  |
| Damages | 19 | All unintended harm or effect in each group (for specific guidance, see "CONSORT for harms") |  |
| **Discussion** | | |  |
| Limitations | 20 | Limitations of the study, addressing sources of possible bias, imprecision and, where appropriate, multiplicity of analyses | 1 |
| Generalization | 21 | Possibility of generalization (external validity, applicability) of trial findings | 1 |
| Interpretation | 22 | Interpretation consistent with the results, with balance of benefits and harms, and considering other relevant evidence | 1 |
| **Other information** | | |  |
| Registration | 23 | Registration number and name of the trial register |  |
| Protocol | 24 | Where the full assay protocol can be accessed, if available |  |
| Financing | 25 | Sources of funding and other support (such as supply of medicines), role of funders |  |

* We strongly recommend reading this checklist together with "the CONSORT 2010 Explanation and Elaboration" to clarify important questions about all items. If applicable, we also recommend reading CONSORT extensions for cluster-randomized trials, non-inferiority and equivalence trials, non-pharmacological treatments, herbal medicine interventions, and pragmatic trials. Other extensions are being prepared: for these and for relevant updated references, related to this checklist, see [www.consort-statement.org](http://www.consort-statement.org)

**12**

**Shek & Yu, 2011**


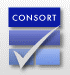
**CONSORT 2010** checklist of information to include when reporting a randomized clinical trial *

| Section/topic | Item No. | Checklist item | Point |
| --- | --- | --- | --- |
| **Title and abstract** | | |  |
|  | 1a | Identified as a randomized trial in the title | 1 |
|  | 1b | Structured summary of trial design, methods, results and conclusions (for specific guidance, see "CONSORT for abstracts") |  |
| **Introduction** | | |  |
| Background and objectives | 2a | Scientific background and justification | 1 |
|  | 2b | Specific objectives or hypotheses | 1 |
| **Methods** |  |  |  |
| Test design | 3a | Description of the assay design (e.g., parallel, factorial), including allocation ratio | 1 |
|  | 3b | Important changes in methods after trial initiation (e.g., selection criteria) and their justification |  |
| Participants | 4a | Participant selection criteria | 1 |
|  | 4b | Origin (centers and institutions) where the data were recorded | 1 |
| Interventions | 5 | Interventions for each group with sufficient detail to allow replication, including how and when they were actually delivered | 1 |
| Results | 6a | A priori specification of primary and secondary response (or outcome) variables, including how and when they were assessed | 1 |
|  | 6b | Any changes in response variables after trial initiation, together with reasons for modification(s) |  |
| Sample size | 7a | How the sample size was determined | 1 |
|  | 7b | If applicable, explain any interim analyses and interruption rules |  |
| Randomization: | | |  |
| Generation of sequenccia | 8a | Method used to generate the random assignment sequence | 1 |
|  | 8b | Type of randomization; Details of any restrictions (such as blocks and block sizes) |  |
| Allocation concealment mechanism | 9 | Mechanism used to implement the random assignment sequence (such as sequentially numbered containers), describing the steps taken to hide the sequence until the interventions were assigned | 1 |
| Implementation | 10 | Who generated the random allocation sequence, who selected participants, and who allocated participants to interventions |  |
| Masking | 11a | If it did, who was blinded after the interventions were allocated (e.g., participants, caregivers, outcome assessors) and how |  |
|  | 11b | If relevant, description of similarity of interventions |  |

| Statistical methods | 12a | Statistical methods used to compare groups in terms of primary and secondary response variables | 1 |
| --- | --- | --- | --- |
|  | 12b | Additional analysis methods, such as subgroup analysis and adjusted analyses | 1 |
| **Results** | | |  |
| Participant flow (a flowchart is strongly recommended) | 13a | For each group, the number of participants who were randomly assigned, who received the proposed treatment, and who were included in the main analysis | 1 |
|  | 13b | For each group, losses and exclusions after randomization, together with the reasons |  |
| Recruitment | 14a | Dates defining recruitment and follow-up periods | 1 |
|  | 14b | Cause of termination or interruption of the trial |  |
| Baseline data | 15 | A table showing baseline demographic and clinical characteristics for each group | 1 |
| Analyzed numbers | 16 | For each group, number of participants (denominator) included in each analysis and whether the analysis was based on the initially assigned groups | 1 |
| Results and estimation | 17a | For each primary and secondary endpoint or outcome, the results for each group, the estimated effect size, and its precision (as 95% confidence interval) | 1 |
|  | 17b | For dichotomous responses, we recommend the presentation of both absolute and relative effect sizes |  |
| Secondary scans | 18 | Results of any other analyses performed, including subgroup analysis and adjusted analyses, differentiating between those specified a priori and exploratory analyses | 1 |
| Damages | 19 | All unintended harm or effect in each group (for specific guidance, see "CONSORT for harms") |  |
| **Discussion** | | |  |
| Limitations | 20 | Limitations of the study, addressing sources of possible bias, imprecision and, where appropriate, multiplicity of analyses | 1 |
| Generalization | 21 | Possibility of generalization (external validity, applicability) of trial findings | 1 |
| Interpretation | 22 | Interpretation consistent with the results, with balance of benefits and harms, and considering other relevant evidence | 1 |
| **Other information** | | |  |
| Registration | 23 | Registration number and name of the trial register |  |
| Protocol | 24 | Where the full assay protocol can be accessed, if available | 1 |
| Financing | 25 | Sources of funding and other support (such as supply of medicines), role of funders |  |

* We strongly recommend reading this checklist together with "the CONSORT 2010 Explanation and Elaboration" to clarify important questions about all items. If applicable, we also recommend reading CONSORT extensions for cluster-randomized trials, non-inferiority and equivalence trials, non-pharmacological treatments, herbal medicine interventions, and pragmatic trials. Other extensions are being prepared: for these and for relevant updated references, related to this checklist, see [www.consort-statement.org](http://www.consort-statement.org)

**23**

**Busch, De Leeuw & Schrijvers, 2013**


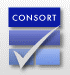
**CONSORT 2010** checklist of information to include when reporting a randomized clinical trial *

| Section/topic | Item No. | Checklist item | Point |
| --- | --- | --- | --- |
| **Title and abstract** | | |  |
|  | 1a | Identified as a randomized trial in the title |  |
|  | 1b | Structured summary of trial design, methods, results and conclusions (for specific guidance, see "CONSORT for abstracts") | 1 |
| **Introduction** | | |  |
| Background and objectives | 2a | Scientific background and justification | 1 |
|  | 2b | Specific objectives or hypotheses |  |
| **Methods** |  |  |  |
| Test design | 3a | Description of the assay design (e.g., parallel, factorial), including allocation ratio |  |
|  | 3b | Important changes in methods after trial initiation (e.g., selection criteria) and their justification |  |
| Participants | 4a | Participant selection criteria |  |
|  | 4b | Origin (centers and institutions) where the data were recorded | 1 |
| Interventions | 5 | Interventions for each group with sufficient detail to allow replication, including how and when they were actually delivered |  |
| Results | 6a | A priori specification of primary and secondary response (or outcome) variables, including how and when they were assessed | 1 |
|  | 6b | Any changes in response variables after trial initiation, together with reasons for modification(s) |  |
| Sample size | 7a | How the sample size was determined |  |
|  | 7b | If applicable, explain any interim analyses and interruption rules |  |
| Randomization: | | |  |
| Generation of sequenccia | 8a | Method used to generate the random assignment sequence |  |
|  | 8b | Type of randomization; Details of any restrictions (such as blocks and block sizes) |  |
| Allocation concealment mechanism | 9 | Mechanism used to implement the random assignment sequence (such as sequentially numbered containers), describing the steps taken to hide the sequence until the interventions were assigned |  |
| Implementation | 10 | Who generated the random allocation sequence, who selected participants, and who allocated participants to interventions |  |
| Masking | 11a | If it did, who was blinded after the interventions were allocated (e.g., participants, caregivers, outcome assessors) and how |  |
|  | 11b | If relevant, description of similarity of interventions |  |

| Statistical methods | 12a | Statistical methods used to compare groups in terms of primary and secondary response variables | 1 |
| --- | --- | --- | --- |
|  | 12b | Additional analysis methods, such as subgroup analysis and adjusted analyses | 1 |
| **Results** | | |  |
| Participant flow (a flowchart is strongly recommended) | 13a | For each group, the number of participants who were randomly assigned, who received the proposed treatment, and who were included in the main analysis |  |
|  | 13b | For each group, losses and exclusions after randomization, together with the reasons |  |
| Recruitment | 14a | Dates defining recruitment and follow-up periods |  |
|  | 14b | Cause of termination or interruption of the trial |  |
| Baseline data | 15 | A table showing baseline demographic and clinical characteristics for each group | 1 |
| Analyzed numbers | 16 | For each group, number of participants (denominator) included in each analysis and whether the analysis was based on the initially assigned groups |  |
| Results and estimation | 17a | For each primary and secondary endpoint or outcome, the results for each group, the estimated effect size, and its precision (as 95% confidence interval) |  |
|  | 17b | For dichotomous responses, we recommend the presentation of both absolute and relative effect sizes |  |
| Secondary scans | 18 | Results of any other analyses performed, including subgroup analysis and adjusted analyses, differentiating between those specified a priori and exploratory analyses | 1 |
| Damages | 19 | All unintended harm or effect in each group (for specific guidance, see "CONSORT for harms") |  |
| **Discussion** | | |  |
| Limitations | 20 | Limitations of the study, addressing sources of possible bias, imprecision and, where appropriate, multiplicity of analyses | 1 |
| Generalization | 21 | Possibility of generalization (external validity, applicability) of trial findings |  |
| Interpretation | 22 | Interpretation consistent with the results, with balance of benefits and harms, and considering other relevant evidence | 1 |
| **Other information** | | |  |
| Registration | 23 | Registration number and name of the trial register |  |
| Protocol | 24 | Where the full assay protocol can be accessed, if available | 1 |
| Financing | 25 | Sources of funding and other support (such as supply of medicines), role of funders |  |

* We strongly recommend reading this checklist together with "the CONSORT 2010 Explanation and Elaboration" to clarify important questions about all items. If applicable, we also recommend reading CONSORT extensions for cluster-randomized trials, non-inferiority and equivalence trials, non-pharmacological treatments, herbal medicine interventions, and pragmatic trials. Other extensions are being prepared: for these and for relevant updated references, related to this checklist, see [www.consort-statement.org](http://www.consort-statement.org)

**11**

**Çelik, 2016**


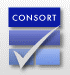
**CONSORT 2010** checklist of information to include when reporting a randomized clinical trial *

| Section/topic | Item No. | Checklist item | Point |
| --- | --- | --- | --- |
| **Title and abstract** | | |  |
|  | 1a | Identified as a randomized trial in the title |  |
|  | 1b | Structured summary of trial design, methods, results and conclusions (for specific guidance, see "CONSORT for abstracts") |  |
| **Introduction** | | |  |
| Background and objectives | 2a | Scientific background and justification | 1 |
|  | 2b | Specific objectives or hypotheses | 1 |
| **Methods** |  |  |  |
| Test design | 3a | Description of the assay design (e.g., parallel, factorial), including allocation ratio | 1 |
|  | 3b | Important changes in methods after trial initiation (e.g., selection criteria) and their justification |  |
| Participants | 4a | Participant selection criteria | 1 |
|  | 4b | Origin (centers and institutions) where the data were recorded |  |
| Interventions | 5 | Interventions for each group with sufficient detail to allow replication, including how and when they were actually delivered | 1 |
| Results | 6a | A priori specification of primary and secondary response (or outcome) variables, including how and when they were assessed | 1 |
|  | 6b | Any changes in response variables after trial initiation, together with reasons for modification(s) |  |
| Sample size | 7a | How the sample size was determined | 1 |
|  | 7b | If applicable, explain any interim analyses and interruption rules |  |
| Randomization: | | |  |
| Generation of sequenccia | 8a | Method used to generate the random assignment sequence | 1 |
|  | 8b | Type of randomization; Details of any restrictions (such as blocks and block sizes) |  |
| Allocation concealment mechanism | 9 | Mechanism used to implement the random assignment sequence (such as sequentially numbered containers), describing the steps taken to hide the sequence until the interventions were assigned |  |
| Implementation | 10 | Who generated the random allocation sequence, who selected participants, and who allocated participants to interventions |  |
| Masking | 11a | If it did, who was blinded after the interventions were allocated (e.g., participants, caregivers, outcome assessors) and how |  |
|  | 11b | If relevant, description of similarity of interventions |  |

| Statistical methods | 12a | Statistical methods used to compare groups in terms of primary and secondary response variables | 1 |
| --- | --- | --- | --- |
|  | 12b | Additional analysis methods, such as subgroup analysis and adjusted analyses | 1 |
| **Results** | | |  |
| Participant flow (a flowchart is strongly recommended) | 13a | For each group, the number of participants who were randomly assigned, who received the proposed treatment, and who were included in the main analysis | 1 |
|  | 13b | For each group, losses and exclusions after randomization, together with the reasons |  |
| Recruitment | 14a | Dates defining recruitment and follow-up periods | 1 |
|  | 14b | Cause of termination or interruption of the trial |  |
| Baseline data | 15 | A table showing baseline demographic and clinical characteristics for each group |  |
| Analyzed numbers | 16 | For each group, number of participants (denominator) included in each analysis and whether the analysis was based on the initially assigned groups | 1 |
| Results and estimation | 17a | For each primary and secondary endpoint or outcome, the results for each group, the estimated effect size, and its precision (as 95% confidence interval) | 1 |
|  | 17b | For dichotomous responses, we recommend the presentation of both absolute and relative effect sizes |  |
| Secondary scans | 18 | Results of any other analyses performed, including subgroup analysis and adjusted analyses, differentiating between those specified a priori and exploratory analyses | 1 |
| Damages | 19 | All unintended harm or effect in each group (for specific guidance, see "CONSORT for harms") |  |
| **Discussion** | | |  |
| Limitations | 20 | Limitations of the study, addressing sources of possible bias, imprecision and, where appropriate, multiplicity of analyses |  |
| Generalization | 21 | Possibility of generalization (external validity, applicability) of trial findings | 1 |
| Interpretation | 22 | Interpretation consistent with the results, with balance of benefits and harms, and considering other relevant evidence | 1 |
| **Other information** | | |  |
| Registration | 23 | Registration number and name of the trial register |  |
| Protocol | 24 | Where the full assay protocol can be accessed, if available |  |
| Financing | 25 | Sources of funding and other support (such as supply of medicines), role of funders | 1 |

* We strongly recommend reading this checklist together with "the CONSORT 2010 Explanation and Elaboration" to clarify important questions about all items. If applicable, we also recommend reading CONSORT extensions for cluster-randomized trials, non-inferiority and equivalence trials, non-pharmacological treatments, herbal medicine interventions, and pragmatic trials. Other extensions are being prepared: for these and for relevant updated references, related to this checklist, see [www.consort-statement.org](http://www.consort-statement.org)

**18**

**de Leeuw, de Bruijn, de Weert-van Oene & Schrijvers, 2010**


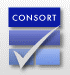
**CONSORT 2010** checklist of information to include when reporting a randomized clinical trial *

| Section/topic | Item No. | Checklist item | Point |
| --- | --- | --- | --- |
| **Title and abstract** | | |  |
|  | 1a | Identified as a randomized trial in the title | 1 |
|  | 1b | Structured summary of trial design, methods, results and conclusions (for specific guidance, see "CONSORT for abstracts") | 1 |
| **Introduction** | | |  |
| Background and objectives | 2a | Scientific background and justification | 1 |
|  | 2b | Specific objectives or hypotheses | 1 |
| **Methods** |  |  |  |
| Test design | 3a | Description of the assay design (e.g., parallel, factorial), including allocation ratio | 1 |
|  | 3b | Important changes in methods after trial initiation (e.g., selection criteria) and their justification |  |
| Participants | 4a | Participant selection criteria |  |
|  | 4b | Origin (centers and institutions) where the data were recorded |  |
| Interventions | 5 | Interventions for each group with sufficient detail to allow replication, including how and when they were actually delivered |  |
| Results | 6a | A priori specification of primary and secondary response (or outcome) variables, including how and when they were assessed | 1 |
|  | 6b | Any changes in response variables after trial initiation, together with reasons for modification(s) |  |
| Sample size | 7a | How the sample size was determined |  |
|  | 7b | If applicable, explain any interim analyses and interruption rules |  |
| Randomization: | | |  |
| Generation of sequenccia | 8a | Method used to generate the random assignment sequence |  |
|  | 8b | Type of randomization; Details of any restrictions (such as blocks and block sizes) |  |
| Allocation concealment mechanism | 9 | Mechanism used to implement the random assignment sequence (such as sequentially numbered containers), describing the steps taken to hide the sequence until the interventions were assigned |  |
| Implementation | 10 | Who generated the random allocation sequence, who selected participants, and who allocated participants to interventions |  |
| Masking | 11a | If it did, who was blinded after the interventions were allocated (e.g., participants, caregivers, outcome assessors) and how |  |
|  | 11b | If relevant, description of similarity of interventions |  |

| Statistical methods | 12a | Statistical methods used to compare groups in terms of primary and secondary response variables | 1 |
| --- | --- | --- | --- |
|  | 12b | Additional analysis methods, such as subgroup analysis and adjusted analyses | 1 |
| **Results** | | |  |
| Participant flow (a flowchart is strongly recommended) | 13a | For each group, the number of participants who were randomly assigned, who received the proposed treatment, and who were included in the main analysis |  |
|  | 13b | For each group, losses and exclusions after randomization, together with the reasons | 1 |
| Recruitment | 14a | Dates defining recruitment and follow-up periods |  |
|  | 14b | Cause of termination or interruption of the trial |  |
| Baseline data | 15 | A table showing baseline demographic and clinical characteristics for each group | 1 |
| Analyzed numbers | 16 | For each group, number of participants (denominator) included in each analysis and whether the analysis was based on the initially assigned groups | 1 |
| Results and estimation | 17a | For each primary and secondary endpoint or outcome, the results for each group, the estimated effect size, and its precision (as 95% confidence interval) | 1 |
|  | 17b | For dichotomous responses, we recommend the presentation of both absolute and relative effect sizes |  |
| Secondary scans | 18 | Results of any other analyses performed, including subgroup analysis and adjusted analyses, differentiating between those specified a priori and exploratory analyses | 1 |
| Damages | 19 | All unintended harm or effect in each group (for specific guidance, see "CONSORT for harms") |  |
| **Discussion** | | |  |
| Limitations | 20 | Limitations of the study, addressing sources of possible bias, imprecision and, where appropriate, multiplicity of analyses | 1 |
| Generalization | 21 | Possibility of generalization (external validity, applicability) of trial findings | 1 |
| Interpretation | 22 | Interpretation consistent with the results, with balance of benefits and harms, and considering other relevant evidence | 1 |
| **Other information** | | |  |
| Registration | 23 | Registration number and name of the trial register |  |
| Protocol | 24 | Where the full assay protocol can be accessed, if available |  |
| Financing | 25 | Sources of funding and other support (such as supply of medicines), role of funders |  |

* We strongly recommend reading this checklist together with "the CONSORT 2010 Explanation and Elaboration" to clarify important questions about all items. If applicable, we also recommend reading CONSORT extensions for cluster-randomized trials, non-inferiority and equivalence trials, non-pharmacological treatments, herbal medicine interventions, and pragmatic trials. Other extensions are being prepared: for these and for relevant updated references, related to this checklist, see [www.consort-statement.org](http://www.consort-statement.org)

**16**

**Du, Jiang & Vance, 2010**


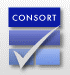
**CONSORT 2010** checklist of information to include when reporting a randomized clinical trial *

| Section/topic | Item No. | Checklist item | Point |
| --- | --- | --- | --- |
| **Title and abstract** | | |  |
|  | 1a | Identified as a randomized trial in the title | 1 |
|  | 1b | Structured summary of trial design, methods, results and conclusions (for specific guidance, see "CONSORT for abstracts") | 1 |
| **Introduction** | | |  |
| Background and objectives | 2a | Scientific background and justification | 1 |
|  | 2b | Specific objectives or hypotheses |  |
| **Methods** |  |  |  |
| Test design | 3a | Description of the assay design (e.g., parallel, factorial), including allocation ratio | 1 |
|  | 3b | Important changes in methods after trial initiation (e.g., selection criteria) and their justification |  |
| Participants | 4a | Participant selection criteria | 1 |
|  | 4b | Origin (centers and institutions) where the data were recorded |  |
| Interventions | 5 | Interventions for each group with sufficient detail to allow replication, including how and when they were actually delivered | 1 |
| Results | 6a | A priori specification of primary and secondary response (or outcome) variables, including how and when they were assessed | 1 |
|  | 6b | Any changes in response variables after trial initiation, together with reasons for modification(s) |  |
| Sample size | 7a | How the sample size was determined | 1 |
|  | 7b | If applicable, explain any interim analyses and interruption rules |  |
| Randomization: | | |  |
| Generation of sequenccia | 8a | Method used to generate the random assignment sequence | 1 |
|  | 8b | Type of randomization; Details of any restrictions (such as blocks and block sizes) |  |
| Allocation concealment mechanism | 9 | Mechanism used to implement the random assignment sequence (such as sequentially numbered containers), describing the steps taken to hide the sequence until the interventions were assigned | 1 |
| Implementation | 10 | Who generated the random allocation sequence, who selected participants, and who allocated participants to interventions |  |
| Masking | 11a | If it did, who was blinded after the interventions were allocated (e.g., participants, caregivers, outcome assessors) and how |  |
|  | 11b | If relevant, description of similarity of interventions |  |

| Statistical methods | 12a | Statistical methods used to compare groups in terms of primary and secondary response variables |  |
| --- | --- | --- | --- |
|  | 12b | Additional analysis methods, such as subgroup analysis and adjusted analyses | 1 |
| **Results** | | |  |
| Participant flow (a flowchart is strongly recommended) | 13a | For each group, the number of participants who were randomly assigned, who received the proposed treatment, and who were included in the main analysis | 1 |
|  | 13b | For each group, losses and exclusions after randomization, together with the reasons | 1 |
| Recruitment | 14a | Dates defining recruitment and follow-up periods |  |
|  | 14b | Cause of termination or interruption of the trial |  |
| Baseline data | 15 | A table showing baseline demographic and clinical characteristics for each group |  |
| Analyzed numbers | 16 | For each group, number of participants (denominator) included in each analysis and whether the analysis was based on the initially assigned groups | 1 |
| Results and estimation | 17a | For each primary and secondary endpoint or outcome, the results for each group, the estimated effect size, and its precision (as 95% confidence interval) | 1 |
|  | 17b | For dichotomous responses, we recommend the presentation of both absolute and relative effect sizes |  |
| Secondary scans | 18 | Results of any other analyses performed, including subgroup analysis and adjusted analyses, differentiating between those specified a priori and exploratory analyses | 1 |
| Damages | 19 | All unintended harm or effect in each group (for specific guidance, see "CONSORT for harms") |  |
| **Discussion** | | |  |
| Limitations | 20 | Limitations of the study, addressing sources of possible bias, imprecision and, where appropriate, multiplicity of analyses |  |
| Generalization | 21 | Possibility of generalization (external validity, applicability) of trial findings | 1 |
| Interpretation | 22 | Interpretation consistent with the results, with balance of benefits and harms, and considering other relevant evidence | 1 |
| **Other information** | | |  |
| Registration | 23 | Registration number and name of the trial register |  |
| Protocol | 24 | Where the full assay protocol can be accessed, if available |  |
| Financing | 25 | Sources of funding and other support (such as supply of medicines), role of funders | 1 |

* We strongly recommend reading this checklist together with "the CONSORT 2010 Explanation and Elaboration" to clarify important questions about all items. If applicable, we also recommend reading CONSORT extensions for cluster-randomized trials, non-inferiority and equivalence trials, non-pharmacological treatments, herbal medicine interventions, and pragmatic trials. Other extensions are being prepared: for these and for relevant updated references, related to this checklist, see [www.consort-statement.org](http://www.consort-statement.org)

**19**

**Hou et al. (2019)**


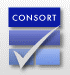
**CONSORT 2010** checklist of information to include when reporting a randomized clinical trial *

| Section/topic | Item No. | Checklist item | Point |
| --- | --- | --- | --- |
| **Title and abstract** | | |  |
|  | 1a | Identified as a randomized trial in the title |  |
|  | 1b | Structured summary of trial design, methods, results and conclusions (for specific guidance, see "CONSORT for abstracts") |  |
| **Introduction** | | |  |
| Background and objectives | 2a | Scientific background and justification | 1 |
|  | 2b | Specific objectives or hypotheses |  |
| **Methods** |  |  |  |
| Test design | 3a | Description of the assay design (e.g., parallel, factorial), including allocation ratio |  |
|  | 3b | Important changes in methods after trial initiation (e.g., selection criteria) and their justification |  |
| Participants | 4a | Participant selection criteria |  |
|  | 4b | Origin (centers and institutions) where the data were recorded | 1 |
| Interventions | 5 | Interventions for each group with sufficient detail to allow replication, including how and when they were actually delivered |  |
| Results | 6a | A priori specification of primary and secondary response (or outcome) variables, including how and when they were assessed | 1 |
|  | 6b | Any changes in response variables after trial initiation, together with reasons for modification(s) |  |
| Sample size | 7a | How the sample size was determined |  |
|  | 7b | If applicable, explain any interim analyses and interruption rules |  |
| Randomization: | | |  |
| Generation of sequenccia | 8a | Method used to generate the random assignment sequence | 1 |
|  | 8b | Type of randomization; Details of any restrictions (such as blocks and block sizes) |  |
| Allocation concealment mechanism | 9 | Mechanism used to implement the random assignment sequence (such as sequentially numbered containers), describing the steps taken to hide the sequence until the interventions were assigned |  |
| Implementation | 10 | Who generated the random allocation sequence, who selected participants, and who allocated participants to interventions |  |
| Masking | 11a | If it did, who was blinded after the interventions were allocated (e.g., participants, caregivers, outcome assessors) and how |  |
|  | 11b | If relevant, description of similarity of interventions |  |

| Statistical methods | 12a | Statistical methods used to compare groups in terms of primary and secondary response variables | 1 |
| --- | --- | --- | --- |
|  | 12b | Additional analysis methods, such as subgroup analysis and adjusted analyses |  |
| **Results** | | |  |
| Participant flow (a flowchart is strongly recommended) | 13a | For each group, the number of participants who were randomly assigned, who received the proposed treatment, and who were included in the main analysis | 1 |
|  | 13b | For each group, losses and exclusions after randomization, together with the reasons |  |
| Recruitment | 14a | Dates defining recruitment and follow-up periods |  |
|  | 14b | Cause of termination or interruption of the trial |  |
| Baseline data | 15 | A table showing baseline demographic and clinical characteristics for each group |  |
| Analyzed numbers | 16 | For each group, number of participants (denominator) included in each analysis and whether the analysis was based on the initially assigned groups | 1 |
| Results and estimation | 17a | For each primary and secondary endpoint or outcome, the results for each group, the estimated effect size, and its precision (as 95% confidence interval) | 1 |
|  | 17b | For dichotomous responses, we recommend the presentation of both absolute and relative effect sizes |  |
| Secondary scans | 18 | Results of any other analyses performed, including subgroup analysis and adjusted analyses, differentiating between those specified a priori and exploratory analyses |  |
| Damages | 19 | All unintended harm or effect in each group (for specific guidance, see "CONSORT for harms") |  |
| **Discussion** | | |  |
| Limitations | 20 | Limitations of the study, addressing sources of possible bias, imprecision and, where appropriate, multiplicity of analyses | 1 |
| Generalization | 21 | Possibility of generalization (external validity, applicability) of trial findings | 1 |
| Interpretation | 22 | Interpretation consistent with the results, with balance of benefits and harms, and considering other relevant evidence |  |
| **Other information** | | |  |
| Registration | 23 | Registration number and name of the trial register |  |
| Protocol | 24 | Where the full assay protocol can be accessed, if available |  |
| Financing | 25 | Sources of funding and other support (such as supply of medicines), role of funders |  |

* We strongly recommend reading this checklist together with "the CONSORT 2010 Explanation and Elaboration" to clarify important questions about all items. If applicable, we also recommend reading CONSORT extensions for cluster-randomized trials, non-inferiority and equivalence trials, non-pharmacological treatments, herbal medicine interventions, and pragmatic trials. Other extensions are being prepared: for these and for relevant updated references, related to this checklist, see [www.consort-statement.org](http://www.consort-statement.org)

**10**
